# *Tropomyosin 1* genetically constrains in vitro hematopoiesis

**DOI:** 10.1186/s12915-020-00783-7

**Published:** 2020-05-14

**Authors:** Christopher Stephen Thom, Chintan D Jobaliya, Kimberly Lorenz, Jean Ann Maguire, Alyssa Gagne, Paul Gadue, Deborah L French, Benjamin Franklin Voight

**Affiliations:** 1 Division of Neonatology, Children’s Hospital of Philadelphia, Philadelphia, PA USA; 2 Department of Systems Pharmacology and Translational Therapeutics, Perelman School of Medicine, University of Pennsylvania, Philadelphia, PA USA; 3 Department of Genetics, Perelman School of Medicine, University of Pennsylvania, Philadelphia, PA USA; 4 Center for Cellular and Molecular Therapeutics, Children’s Hospital of Philadelphia, Philadelphia, PA USA; 5 Department of Pathology and Laboratory Medicine, Children’s Hospital of Philadelphia, Philadelphia, PA USA; 6 Institute of Translational Medicine and Therapeutics, University of Pennsylvania, Philadelphia, PA USA

**Keywords:** Hematopoiesis, Genetics, Tropomyosin 1, Induced pluripotent stem cells

## Abstract

**Background:**

Identifying causal variants and genes from human genetic studies of hematopoietic traits is important to enumerate basic regulatory mechanisms underlying these traits, and could ultimately augment translational efforts to generate platelets and/or red blood cells in vitro. To identify putative causal genes from these data, we performed computational modeling using available genome-wide association datasets for platelet and red blood cell traits.

**Results:**

Our model identified a joint collection of genomic features enriched at established trait associations and plausible candidate variants. Additional studies associating variation at these loci with change in gene expression highlighted *Tropomyosin 1* (*TPM1*) among our top-ranked candidate genes. CRISPR/Cas9-mediated *TPM1* knockout in human induced pluripotent stem cells (iPSCs) enhanced hematopoietic progenitor development, increasing total megakaryocyte and erythroid cell yields.

**Conclusions:**

Our findings may help explain human genetic associations and identify a novel genetic strategy to enhance in vitro hematopoiesis. A similar trait-specific gene prioritization strategy could be employed to help streamline functional validation experiments for virtually any human trait.

## Introduction

Elucidating genetic mechanisms governing hematopoiesis has broad value in understanding blood production and hematologic diseases [1]. Given interest in generating platelets and red blood cells (RBCs) from in vitro culture of induced pluripotent stem cells (iPSCs) [2–4], there is also translational value in harnessing genetic and molecular processes that regulate hematopoiesis. Cost-effective blood cell generation will require novel strategies based on better knowledge of underlying mechanisms driving in vitro development.

In vitro hematopoietic systems might be improved by identifying novel factors from human genetic studies. Genome-wide association studies (GWAS) have linked hundreds of single nucleotide polymorphisms (SNPs) with platelet and/or red cell trait variability [5, 6]. Because most GWAS SNPs are non-coding, likely influencing transcriptional expression of key genes [7, 8], it has been challenging to derive functional biochemical understanding of the key genes of action [8–10]. Relatively few studies have elucidated biochemical mechanisms for blood trait variability loci [11–15]. One strategy to narrow focus on candidate genes is to link non-coding variation to expression of nearby genes [1, 16, 17]. However, for platelet trait variation alone, GWAS have thus far implicated > 6700 expression quantitative trait loci (eQTLs) affecting expression of > 1100 genes (see the “Methods” section). Hence, there is a clear need to more specifically identify putatively functional sites.

Actin cytoskeletal dynamics play key roles in hematopoiesis [18–20]. Tropomyosin proteins coat most actin filaments and regulate actin functions [21, 22]. All four human tropomyosin genes (1–4) are expressed in human hematopoietic cells, and *Tropomyosin 4* promotes platelet development [15]. Genetic studies have also suggested a role for *Tropomyosin 1* (*TPM1*) in human platelet trait variation [6], though no prior studies have elucidated if or how *TPM1* impacts human hematopoiesis.

Here, we utilized penalized regression to construct a model that predicted platelet GWAS associations based on epigenetic datasets as features for the prediction. Our model built from platelet trait GWAS loci reliably distinguished sentinel GWAS SNPs, as well as platelet-relevant genes and loci. Among these prioritized sites were SNPs that regulate *TPM1* expression. To validate this putative candidate gene and to explore its function, we used CRISPR/Cas9 genome editing to discover that cultured *TPM1*-deficient induced pluripotent stem cells enhanced hematopoietic progenitor cell formation. In turn, this increased functional megakaryocyte (MK) yield. Thus, our framework stratified relevant functional loci and helped identify *TPM1* manipulation as a novel strategy to enhance in vitro hematopoiesis.

## Results

### Penalized regression model identifies genetic regulatory loci for hematopoiesis

**Fig. 1.**
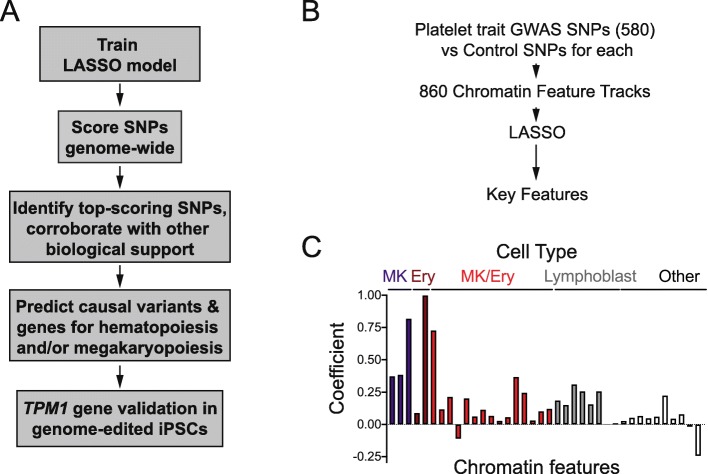
A penalized regression-based approach to identify hematopoietic regulatory loci and genes. **a** Schematic outline of our approach. We generated a penalized regression-based predictive scoring algorithm based on platelet trait GWAS loci and applied the resultant scoring algorithm genome-wide to predict causal variants and genes. We validated this model computationally and through validation of *TPM1* function in induced pluripotent stem cells (iPSCs). **b** To generate a penalized regression model, 580 platelet trait GWAS SNPs [6] and matched control SNPs (~ 100 per GWAS SNP [25]) were analyzed for overlap with 860 chromatin features (e.g., histone marks and transcription factor binding sites). **c** Penalized regression (LASSO [23]) analysis identified 38 chromatin features from the indicated cell types that best discriminated GWAS SNPs, after controlling for background features (distance to nearest gene, number of SNPs in linkage disequilibrium, and minor allele frequency). Bar heights are LASSO coefficients, indicating the relative importance of each feature. MK, primary megakaryocytes; Ery, peripheral blood derived erythroblasts; MK/Ery, K562 cells; Lymphoblast, GM12878 or GM12891

GWAS have linked hundreds of single nucleotide polymorphisms (SNPs) with variability in human platelet traits [6]. To focus our studies on credible functional follow-up candidates, we utilized a penalized logistic regression framework, i.e., the least absolute shrinkage and selection operator (LASSO) [23, 24]. We used 860 features to construct a model that distinguished platelet trait GWAS SNPs from control SNPs after controlling for allele frequency, distance to gene, and number of SNP proxies in strong linkage disequilibrium (Fig. 1a, the “Methods” section, and Additional file 1: Table S1).

Our “platelet trait model” was trained on 580 genome-wide-significant platelet trait-related SNPs from a large recent GWAS of human blood trait variation [6], along with 860 chromatin features (Fig. 1b). These GWAS SNPs affected human platelet count (PLT), platelet-crit (PCT), mean platelet volume (MPV), and/or platelet distribution width (PDW). For each GWAS SNP, we identified control SNPs matched to the degree possible on distance to nearest gene, number of SNPs in linkage disequilibrium, and minor allele frequency. We forced our models to include these background characteristics, in order to ensure that we identified chromatin features that would distinguish GWAS SNPs after controlling for background genetic variables. Model performance in the training phase was assessed using standard approaches (i.e., 10-fold cross-validation).

The resultant predictive model comprised 38 epigenomic features that best distinguished platelet trait GWAS SNPs from controls (Fig. 1c, Additional file 2: Figure S1, and Additional file 1: Table S2). Background features were included during model creation and are reflected in the area under the receiver operator curve (AUC) for the initial training phase. However, given our interest in genomic positions and overlapping chromatin features, background characteristics were not carried forward for genome-wide model application. These background characteristics would not affect determination of human trait-associated loci based on genomic context.

While some care in interpretation was required, it was encouraging that the model selected biologically plausible features. GATA1, GATA2, SCL, and FLI1 are critical hematopoietic transcription factors [26, 27], and most of our features came from hematopoietic cell types (primary MK, peripheral blood-derived erythroblasts, K562 with MK/erythroid potential, and GM12878/GM12891 lymphoblasts; Additional file 1: Table S2).

### Genome-wide model application

**Fig. 2.**
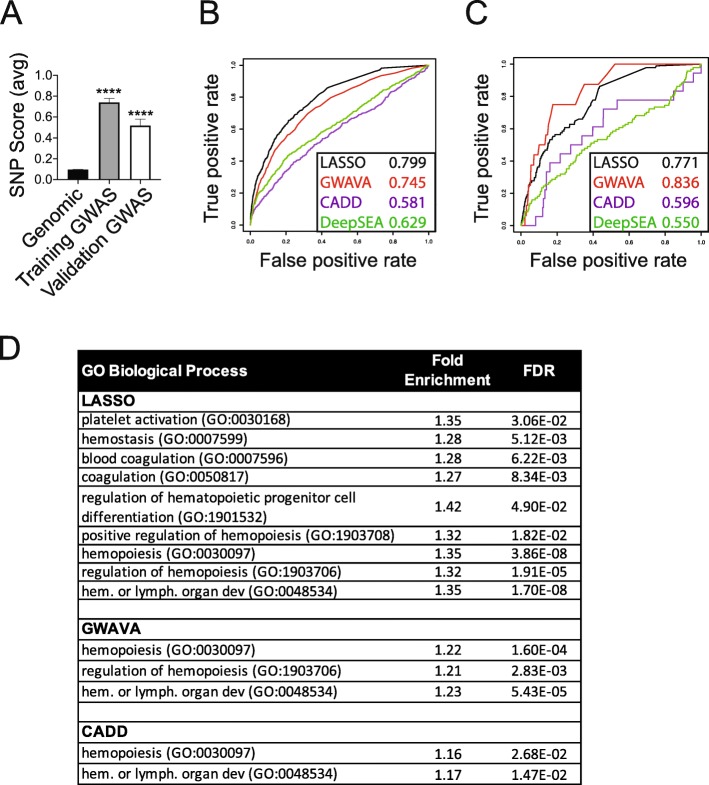
Penalized regression model identifies genes relevant to platelet and hematopoietic biology. **a** SNP scores for platelet model training SNPs, or a set of validation platelet trait SNPs, were significantly higher than genome-wide SNP scores. Bars represent mean ± SEM, ****p < 0.0001 by ANOVA. **b** Performance comparison of our platelet trait model to DeepSEA [28], GWAVA [17], and CADD [29] for training platelet trait SNP identification. AUC values are shown in the legend. **c** Performance comparison for validation platelet trait SNP identification. There was substantial variation in the number of validation SNPs recognized and scored by each model. AUC values shown in the legend represent prediction accuracy in identifying validation SNPs for LASSO (*n* = 94 SNPs), GWAVA (*n* = 16), CADD (*n* = 18), and DeepSEA (*n* = 94) vs ~ 10,000 random control SNPs. **d** Platelet and hematopoiesis pathways [30] identified by the highest-scoring (top 1%) SNPs genome-wide for the indicated models, excluding established platelet trait loci [6] (FDR, false discovery rate)

We calculated trait-enrichment scores genome-wide based on SNP overlap with each of the selected chromatin features, weighted by our penalized regression model coefficients (see the “Methods” section and Additional file 1: Table S2). As expected, training SNP scores were significantly higher for platelet trait GWAS SNPs relative to SNPs genome-wide (*p* < 0.0001 by ANOVA, Fig. 2a). A set of 94 validation platelet trait GWAS SNPs, representing 15% of all platelet trait GWAS SNPs [6], also scored significantly higher than genome-wide SNPs, although not as well as training SNPs (Fig. 2a).

### Application of additional prediction methods

Our goal was to use a compilation of methods and evidence to specify loci with high functional likelihood. Some models have been previously developed to identify active genomic loci (e.g., CADD [29], GWAVA [17], and DeepSEA [28]). We compared the effectiveness of these models, and our trait-specific model, to discriminate training or validation platelet trait GWAS sites from sets of ~ 100 control SNPs for each GWAS SNP. LASSO scores were based only on overlaps with chromatin features and associated coefficients. We used AUC values to assess model performance. Our trait-specific model performed well in analyses of training SNPs (AUC 0.799, Fig. 2b) and validation SNPs (AUC 0.771, Fig. 2c). GWAVA also performed well in predicting training SNPs (AUC 0.745, Fig. 2b) and validation SNPs (AUC 0.836, Fig. 2c).

GWAVA prioritizes functional impact of non-coding genomic elements without regard for lineage or trait specificity [17]. Hence, our results suggested that chromatin marks associated with active gene regulatory regions were enriched in platelet trait GWAS loci.

However, hematopoiesis- and blood lineage-specific chromatin regulatory mechanisms are also critical for blood development [31–33]. It was difficult to parse hematopoietic biological rationale in the regulatory elements prioritized by GWAVA scoring. Therefore, we pursued further validation of our trait-specific model, in an effort to best specify loci and related genes that were important for hematopoiesis, megakaryopoiesis, and/or platelet biology.

### Genome-wide model validation

Encouraged by the features we selected and our model performance, we next sought to derive external support for the model selected by our regression framework. First, we evaluated the biological specificity of variation prioritized by the model. This was particularly important, given practical limitations associated with fine-mapping and cellular validation experiments. Gene Ontology analysis of the top 1% highest-scoring SNPs indicated that the nearest genes to penalized regression-prioritized variants were enriched for biologically relevant pathways, even after removing GWAS-significant sites (Fig. 2d and Additional file 1: Table S3-S5). While many associated pathways related to platelet function and coagulation, generalized hematopoiesis- and hematopoietic progenitor cell-related pathways were also included.

Second, we asked whether our SNP scores correlated with summary association statistics for platelet trait-GWAS data [6]. Indeed, variants that were nominally associated with platelet traits but did not reach genome-wide significance and not included in our model (*p* value between 0.05 and 5 × 10^−8^) had significantly higher average scores compared to SNPs that were not obviously associated (*p* value > 0.05, Additional file 2: Figure S2). This correlation suggested that our scoring algorithm was valid genome-wide and could potentially reveal true biological associations, as had the GWAS [5, 11, 12, 14].

Finally, we asked if regulatory gene enhancer regions were enriched with high-scoring SNPs by our model, consistent with regulatory function. We found that our model assigned higher scores to SNPs in FANTOM5 enhancer regions [34] compared with other sites genome-wide, consistent with the hypothesis that functional non-coding SNPs associate with active regulatory regions [8, 35] (Additional file 2: Figure S3, enhancer region scores > 0.9 vs genome-wide baseline < 0.1). We further observed that enhancer regions in hematopoietic cell types scored significantly higher than enhancers from irrelevant control cells (Additional file 2: Figure S3). These data suggest trait specificity in hematopoietic enhancers, consistent with prior studies [31], and the broader hypothesis about tissue-specific trait heritability as reported elsewhere [36, 37]. Collectively, our findings indicated that we could successfully target hematopoietic and platelet trait-relevant loci.

### Exemplary candidate locus and gene identification

Next, we used computational predictions, including our own model, to stratify sites and related genes for functional validation. Given practical limitations related to follow-up validation, we wanted to narrow our focus to a modest number of loci (e.g., < 20). We reasoned that functional SNPs would (i) be in high linkage disequilibrium (LD) with established platelet trait GWAS loci, (ii) score highly relative to other SNPs within that LD block, (iii) regulate target gene(s) as expression quantitative trait loci (eQTLs), and (iv) overlap GATA binding sites [38, 39]. We prioritized GATA binding sites based on the importance of GATA factors in hematopoiesis [26, 40] and in our penalized regression model (Additional file 1: Table S2). We specifically focused our attention on sites that were scored in the top 5% genome-wide by our platelet trait model and by GWAVA [17], a more generalized machine learning-based model that performed well in validation analyses (Fig. 2b, c).

This stratification approach identified 15 loci and related genes, including SNPs known to impact hematopoiesis, megakaryocyte, and/or platelet biology (Table 1 and Additional file 2: Figure S4). In principle, *any* site meeting these stringent criteria could form the basis for interesting biological follow-up experiments.

**Table 1:** Penalized regression-based fine-mapping identifies eQTLs in established platelet trait GWAS loci that overlie GATA binding sites. Listed SNPs are within platelet trait GWAS LD blocks (EUR *r*^2^ > 0.7), scored in the top 5% by our platelet trait model and by GWAVA [17], overlap canonical or near-canonical GATA binding sites, and are eQTLs for at least 1 gene [41] (GTEx V7). Associated gChromVAR posterior probabilities of being causal for platelet count trait association (PP PLT) are shown [1]. Genes in boldface have known hematopoietic function. SNP rsIDs and locations refer to hg19 genome

rsID	Chr	Pos (Mb)	Platelet score (percentile)	GWAVA score (percentile)	gChromVAR (PP PLT)	Nearest gene	eQTL gene(s)
rs11240368	1	205.1	1.12 (97th)	0.52 (95th)		*DSTYK*	*CNTN2*, *TMEM81*
rs3771535	2	70.0	0.94 (95th)	0.53 (95th)	0.01	*ANXA4*	*GMCL1*, *SNRNP27*
rs10180681	2	121.0	1.41 (98th)	0.63 (97th)	0.01	*RALB*	*EPB41L5*, ***PTPN4*** [42], ***RALB*** [43]
rs10180682	2	121.0	1.41 (98th)	0.64 (97th)	0.01	*RALB*	*EPB41L5*, ***PTPN4*** [42], ***RALB*** [43]
rs9646785	2	172.0	1.27 (98th)	0.58 (96th)		*TLK1*	*GAD1*, *GORASP2*
rs6771578	3	167.4	1.14 (97th)	0.60 (96th)	0.003	*PDCD10*	***PDCD10*** [44], *SERPINI1*, *WDR49*
rs12652692	5	77.8	3.62 (99th)	0.57 (96th)	0.01	*LHFPL2*	*LHFPL2*, *SCAMP1*
rs72793280	5	131.6	2.73 (99th)	0.89 (99th)	0.001	*P4HA2*	*ACSL6*, ***P4HA2*** [45, 46], ***PDLIM4*** [47], *SLC22A4*, *SLC22A5*
rs1741820	6	122.8	1.75 (99th)	0.55 (96th)		*HSF2*	*HSF2*, *PKIB*
rs342293	7	106.4	1.80 (99th)	0.94 (99th)	0.99	*CC71L*	***PIK3CG*** [12]*
rs13265995	8	56.7	1.75 (99th)	0.60 (96th)		*TMEM68*	***LYN*** [48, 49], *TGS*, *TMEM68*
rs9704108	11	0.3	1.12 (97th)	0.85 (99th)	0.087	*IFITM2*	*IFITM2*
rs11071720	15	63.3	1.53 (98th)	0.58 (96th)	0.98	*TPM1*	*APH1B*, *LACTB*, *RAB8B*, ***TPM1*** [5]**
rs2316513	17	2.0	0.92 (95th)	0.54 (95th)	0.005	*EST1A*	*DPH1*, *SMG6*, ***SRR*** [50]
rs1654439	19	55.6	2.69 (99th)	0.58 (96th)	0.002	*RDH13*	***GP6*** [49, 51], *NLRP2*, ***RDH13*** [52]

*eQTL in human platelets [12], but not in GTEx tissues [41]

**Function suggested by *D. rerio* morpholino experiments [5]

**Fig. 3.**
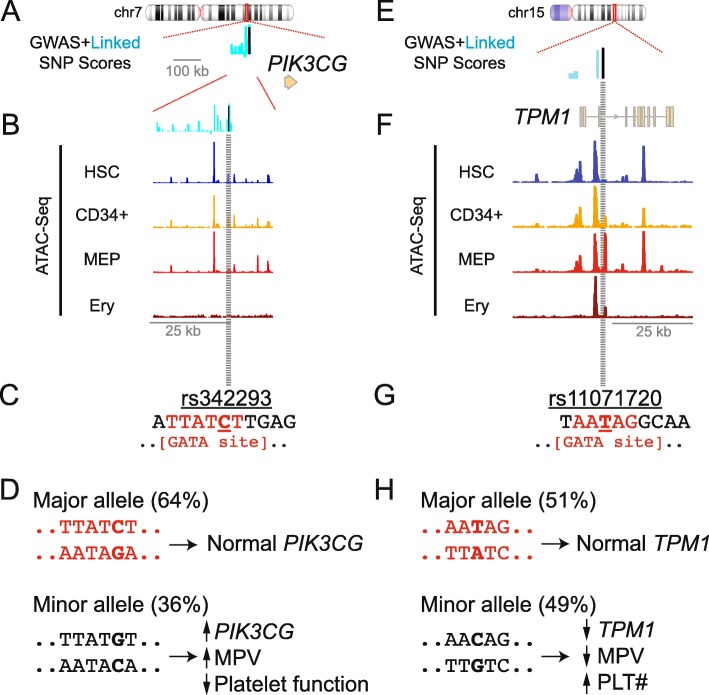
Exemplary high-scoring eQTLs near the *PIK3CG* and *TPM1* gene loci overlap putative GATA binding sites and are associated with altered platelet traits. **a** High-scoring platelet trait GWAS SNP rs342293 (black) and linked SNPs (EUR *r*^2^ > 0.7, cyan) lie upstream of *PIK3CG*. Bar heights depict SNP scores. **b** This region overlaps a dynamic accessible chromatin region during hematopoiesis [53]. Accessible chromatin (ATAC-Seq) data are shown for hematopoietic stem cells (HSC), CD34+ hematopoietic progenitor cells, megakaryocyte-erythroid progenitors (MEP), and erythroblasts (Ery). **c** The local DNA sequence for rs342293 (underlined) includes a canonical GATA binding site [38] (red). **d** Platelet phenotypes associated with rs342293 alleles [12]. **e** SNP scores near platelet trait GWAS SNP rs11071720 (black) and linked SNPs (EUR *r*^2^ > 0.7, cyan). Bar heights depict SNP scores. **f** ATAC-Seq regions at this locus are shown for the indicated cell types [53]. **g** Local DNA sequence shows a putative GATA binding site [38] (red) around rs11071720 (underlined “T”). **h** The major and minor rs11071720 alleles and associated platelet phenotypes [6, 54]. Allele percentages based on UCSC Genome Browser and dbSNP

Two of these loci stood out as high-scoring variants by the recently described gChromVAR algorithm [1], which is based on accessible chromatin regions in hematopoietic cells (Table 1). First, rs342293 is a GWAS SNP [5] that regulates *PIK3CG* gene expression [12] and lies within accessible chromatin in hematopoietic progenitor cell types [53] (Fig. 3a, b). The GATA site is disrupted in the presence of the SNP minor allele (Fig. 3c). In platelets, *PIK3CG* activity regulates PIK3 signaling [55] and response to collagen [56]. Individuals harboring this minor allele had increased MPV and decreased platelet reactivity [12] (Fig. 3d).

A second variant, rs11071720, found within the 3rd intron of the *Tropomyosin 1* (*TPM1*) gene locus, also attracted our attention. This sentinel GWAS SNP scored highly compared to linked SNPs (EUR *r*^2^ > 0.7) and overlapped accessible chromatin in hematopoietic cells [53] (Fig. 3e, f). The rs11071720 minor allele, which disrupts a near-canonical GATA binding site, is an eQTL associated with decreased *TPM1* expression [41, 54], higher platelet count, and lower MPV [6] (Fig. 3g, h and Additional file 2: Figure S5).

Tropomyosin proteins regulate actin cytoskeletal functions, which are critical for hematopoietic, megakaryocyte, and platelet biology [15, 19, 20, 57]. Although morpholino studies showed *TPM1* to be important for zebrafish thrombopoiesis [5], no prior study had examined the effect of *TPM1* during human hematopoiesis. Based on these and the human genomics data, we hypothesized that *TPM1* would be an important effector of hematopoiesis and ultimately platelet biology. Thus, in what follows, we focus our cellular validation studies on *TPM1*, under the hypothesis that rs11071720 regulated the expression of this gene.

### *Tropomyosin 1* modulation enhances in vitro hematopoiesis

**Fig. 4.**
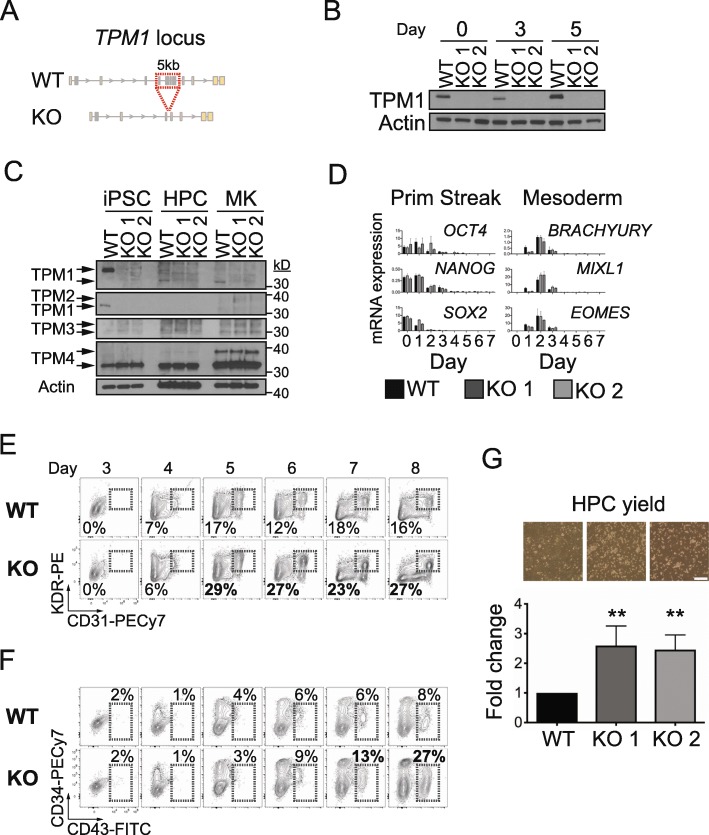
*TPM1* deficiency enhances iPSC-derived hematopoietic progenitor cell formation. **a** A 5-kb region (*TPM1* exons 4–8, red box) was targeted for CRISPR/Cas9-mediated deletion to create KO iPSCs. **b** Western blots showing TPM1 protein expression at the indicated differentiation days. **c** Western blots showing TPM1–4 expression in wild type (WT) and KO iPSCs, hematopoietic progenitor cells (HPC, differentiation d8), and FACS-sorted MKs (CD41^+^/CD42b^+^, expansion d3). TPM1 antibodies targeted exon 4 (top) or exon 9d (2nd panel). **d** Primitive streak and mesoderm gene expression are normal in differentiating KO iPSCs. **e**, **f** KO cells yield **e** KDR^+^/CD31^+^ cells and **f** CD43^+^ hematopoietic progenitor cells (HPC) with normal kinetics, but in enhanced abundance. Percent (%) cells within boxed regions for WT and KO clone 2 are shown from a representative experiment. **g** Quantification of WT and KO non-adherent HPCs on differentiation day 8. Bars represent fold change in HPCs (mean ± SD) vs WT for ≥ 4 experiments. **p < 0.01 by ANOVA. (Top) Culture images on differentiation d8, with HPCs (light color) floating above an adherent monolayer. Scale bar, 20 mm

We investigated functions for the *TPM1* gene in an in vitro human model of primitive hematopoiesis [58]. We expected that total gene deletion would show stronger effects than non-coding SNP modification [59]. Using CRISPR/Cas9, we targeted a ~ 5-kb region containing *TPM1* exons 4–8 in iPSCs (Fig. 4a), anticipating creation of a null allele [60]. We confirmed deletion by sequencing and western blot (Fig. 4b, c and Additional file 2: Figure S6). In total, we obtained 3 *TPM1* knockout (KO) clones from 2 separate genetic backgrounds. Karyotype and copy number variation analyses confirmed that engineering these clones did not introduce any de novo genomic aberrancies (Additional file 1: Table S6 and Additional file 2: Figure S7).

TPM1 protein was present during early iPSC differentiation, but downregulated in non-adherent hematopoietic progenitor cells and differentiated MKs (Fig. 4b, c). Early differentiation proceeded normally in KO clones, with normal patterns of primitive streak and mesoderm gene expression (Fig. 4d), as well as pluripotency marker loss (Additional file 2: Figure S8). The kinetics by which KDR^+^/CD31^+^ endothelial/hemogenic endothelial cells and CD43^+^ hematopoietic progenitor cells (HPCs) emerged were also normal (Fig. 4e, f). In this culture system, KDR^+^/CD31^+^ cells include both HPC precursor cells (hemogenic endothelium) as well as cells destined for a purely endothelial fate.

Unexpectedly, we found that KO cultures enhanced generation of KDR^+^/CD31^+^ as well as CD43^+^ HPCs (Fig. 4e, f). We quantified HPC abundance by cell counting and flow cytometry, observing that KO HPC yield doubled that of WT controls (Fig. 4g). We confirmed this finding in a KO clone from a genetically distinct iPSC background (Additional file 2: Figure S9). All HPCs retained normal hematopoietic cell surface marker expression (Additional file 2: Figure S10).

**Fig. 5.**
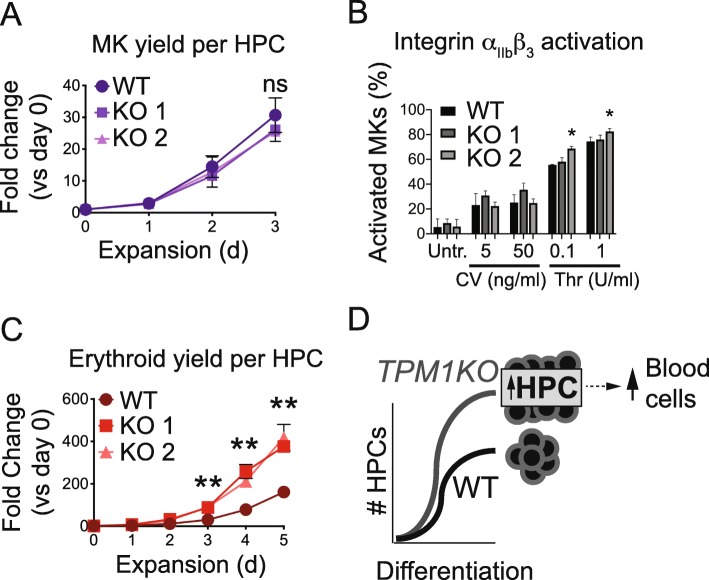
*TPM1*-deficient hematopoietic progenitor cells yield normal-to-increased quantities of functional megakaryocytes and erythroid cells. **a** WT and KO HPCs put into MK expansion culture generate equivalent numbers of MKs. Points represent CD41^+^/CD42b^+^ MK percentage multiplied by total cell count, normalized to cell count on day 0. ns, not significant. **b***TPM1* KO MKs respond appropriately to platelet agonists. WT and KO MKs were incubated with Convulxin (CV) or Thrombin (Thr) at the indicated concentrations, and the percentage of activated MKs (PAC-1^+^/CD41^+^/CD42b^+^) were quantified. *p < 0.05 by ANOVA vs WT. **c** KO HPCs put into erythroid expansion culture generate more erythroid cells than WT HPCs. Points represent CD235^+^ percentage multiplied by total cell count, normalized to cell count on day 0. **p < 0.01 by ANOVA. **d** Model in which KO iPSCs yield more HPCs than WT, generating more total blood cells

Next, we investigated whether KO HPCs would yield functional megakaryocytes in increased quantities. Liquid expansion culture revealed normal mature CD41^+^/CD42b^+^ megakaryocyte yield per HPC (Fig. 5a). With twice as many starting HPCs, this meant that total megakaryocyte recovery increased ~ 2-fold in KO cultures. KO megakaryocyte morphology was normal (Additional file 2: Figure S11), and megakaryocyte activation in response to agonists was normal-to-increased (Fig. 5b). Microarray gene expression analyses of WT and KO megakaryocytes revealed no statistically significant changes in megakaryocyte genes (Additional file 2: Figure S12 and Additional file 1: Table S7).

The early hematopoietic phenotype in KO cultures was unexpected. We asked whether KO HPCs might also enhance yield of other blood cell types. Indeed, KO HPCs spawned normal-to-increased quantities of erythroid and myeloid cells (Fig. 5c and Additional file 2: Figure S13). Hence, *TPM1* deletion enhanced formation of HPCs with multilineage potential (Fig. 5d).

### *Tropomyosin 1* locus is prioritized by red cell trait-based penalized regression model

We were surprised by the early hematopoietic effects of *TPM1* deletion, given that rs11071720 has only been genetically linked with platelet traits [6]. We therefore investigated whether this finding could have been predicted using human genetics data. We found that rs11071720-linked regulatory variants were marginally associated with red cell traits, although these data did not meet genome-wide significance (Additional file 2: Figure S14). It is possible that future studies with improved power will reveal a true statistical association with red cell traits at this locus.

We also trained an additional model for red cell traits, using an analogous framework and regulatory features as described for platelet traits (see the “Methods” section). Model training used 818 red blood cell trait-related GWAS SNPs affecting red blood cell count (RBC count), hematocrit (HCT), mean red cell corpuscular volume (MCV), and/or red cell distribution width (RDW). The resultant model included 78 features and performed well in distinguishing red cell trait GWAS SNPs (Additional file 2: Figure S15 and Additional file 1: Table S8). When used as a scoring algorithm genome-wide, this red cell trait model displayed performance similar to the platelet trait model (Additional file 2: Figure S16, S17 and Additional file 1: Table S9).

Interestingly, our red cell model scored rs11071720 in the 96th percentile genome-wide (Additional file 1: Table S10). This prioritization agrees with *TPM1* impacting both megakaryocyte and erythroid lineages. The other 14 sites that scored in the top 5% by both platelet and red cell models might also be expected to regulate early hematopoietic biology, and could form the basis for future cellular validation experiments (Additional file 1: Table S10). Indeed, several of these genes are known to regulate hematopoiesis.

## Discussion

Genetic insights could augment efforts to generate blood products in vitro [2–4], but relatively few genetically implicated loci or genes have been functionally validated [11–15]. The purposes of our present study were to establish (i) whether computational approaches using available genomic data could prioritize trait-specific sites and genes that impact hematopoiesis, megakaryopoiesis, and/or platelet biology, and (ii) to validate the function of a novel candidate gene (i.e., *TPM1*) in a translationally relevant iPSC model. Our data support a model whereby *TPM1* deficiency enhances in vitro formation of multilineage HPCs (Fig. 5d). In addition to understanding a genetic modifier of hematopoietic traits [6], application of our results may augment in vitro megakaryocyte and erythroid cell yields.

Broadly, the successful implementation of this trait-specific penalized regression method demonstrates a tunable approach to variant and gene identification. Our pipeline is similar to prior methods that have stratified loci based on chromatin feature data (e.g., GWAVA [17] and fGWAS [61]), but is readily scalable to any set of loci and chromatin features. For blood-related traits, it is an adaptable complement to established and excellent scoring models such as gChromVAR [1].

Given the scope of the present study, the most important functional result was enhanced yield of HPCs and functional megakaryocytes. Our results were directionally consistent with human genetic data [6], finding that decreased *TPM1* expression portends higher megakaryocyte yield. The molecular mechanism(s) driving enhanced hemogenic endothelium and/or HPC formation will be of considerable biologic and translational interest, and such studies are ongoing. *TPM1* KO-related increases in HPC formation may complement or synergize previously described approaches that enhanced later stages of hematopoiesis [2, 3, 62, 63].

Early hematopoietic function for *TPM1* was unexpected based on blood genetics [6]. Our model may have prioritized some “early” hematopoietic sites, given that many chromatin features derived from relatively immature megakaryocytes [26] as well as K562 cells, which can act as progenitors for erythroid or megakaryocyte lineages. Indeed, some of the sites targeted general hematopoietic- and HPC-related pathways (Fig. 2d). Chromatin feature data from mature megakaryocytes may enable future models to more specifically target late stage megakaryopoiesis and/or platelet sites. Alternatively, *TPM1* could have separate functions in early and late hematopoiesis, akin to *GATA2* [64].

Though a lack of robust detection methods precluded accurate platelet production quantitation in our culture system, normal function of derived megakaryocytes suggests an overall increase in megakaryocyte yield would translate into higher platelet production. Importantly, our findings do not exclude additional effects on terminal megakaryopoiesis or erythroid development in vitro, nor in vivo effects outside the scope of our iPSC model.

Enhanced hematopoiesis in *TPM1KO* iPSCs contrasts detrimental effects of *TPM1* deficiency on organism fitness in other contexts [5, 65, 66]. For example, abrogated *D. rerio* thrombopoiesis with *tpma*-directed morpholinos [5] resembles human *TPM4* deficiency [15] rather than *TPM1* deficiency. This highlights the importance of species-specific genetic validation, particularly given inter-species disparities in hematopoiesis [67].

## Conclusions

In conclusion, using a penalized regression modeling approach to functional variant identification led us to define a role for *TPM1* in constraining in vitro hematopoiesis. Recent advances increasing per-MK platelet yields [2] have focused a spotlight on increasing cost-effectiveness of earlier stages of in vitro hematopoiesis. In addition to improved recognition of genes and mechanisms underlying quantitative hematopoietic trait variation, application of the computational approach described herein could also help to specify trait-specific causal genetic variants for virtually any clinically relevant human trait.

## Methods

### In silico analyses

Relevant datasets and coding scripts can be found on GitHub (https://github.com/thomchr/2019.PLT.TPM1.Paper). Human genome version hg19 was used for all analyses, and we utilized the LiftOver script when necessary (https://bioconductor.org/packages/release/workflows/html/liftOver.html). GWAS summary statistics are publicly available (http://www.bloodcellgenetics.org/).

### Expression quantitative trait locus analysis

To estimate the number of eQTLs implicated by prior platelet trait GWAS, SNPs in high LD with established GWAS loci [6] (EUR *r*^2^ > 0.9) were identified using PLINK. From this set of SNPs, eQTLs and affected genes were identified from GTEx V7 [41]. Numbers reported in the text reflect unique eQTL SNPs, which often functioned across multiple tissues. The affected gene estimate reflects the number of unique Ensembl gene identifiers (ENSG).

### SNP selection

From a total of 710 genome-wide significant GWAS SNPs (*p* < 5E−8) affecting platelet count, platelet-crit, mean platelet volume, and/or platelet distribution width [6], 580 comprised our platelet model training SNP set. These 580 had rsIDs that were recognized by the Genomic Regulatory Elements and GWAS Overlap algoRithm (GREGOR) [25] tool, which we used to select control SNPs based on distance to nearest gene, number of SNP LD proxies linked to the lead associated SNP (*r*^2^ ≥ 0.8), and minor allele frequency. We identified ~ 100 matched controls for each training SNP, all with a minor allele frequency > 10%. This minor allele cutoff was necessary to limit the effects of very low control SNP frequencies on the resultant model.

From a total of 1003 genome-wide significant GWAS SNPs (*p* < 5E−8) affecting red cell count, hematocrit, mean corpuscular volume, and/or red cell distribution width [6], 818 had rsIDs recognized by GREGOR. These comprised the red cell model training SNP set. We identified ~ 100 matched controls with minor allele frequency > 10% for each training SNP.

### Chromatin feature selection

We collected a subset of available feature tracks from ENCODE [68], including data for hematopoietic (K562, GM12878, and GM12891) as well as other cell types (e.g., H1-hESC, HUVEC, HeLa, HepG2). We also collected available feature tracks from primary MKs and hematopoietic cells [26]. The only modification to any of these genomic datasets was peak-calling in MK-derived chromatin immunoprecipitation-sequencing (ChIP-Seq) tracks [69]. See Additional file 1: Table S1 for a list of these features.

### Penalized regression modeling

To generate our model, we first analyzed training set GWAS SNPs and matched control SNPs for overlap with 860 chromatin features (dataset available on GitHub). Columns representing our 3 baseline parameters (distance to nearest gene, number of LD proxies linked to the lead associated SNP, and minor allele frequency) were also included in this data table for each SNP. This chromatin feature overlap data file was then analyzed using the least absolute shrinkage and selection operator (LASSO, L1 regularization, glmnet version 2.0-18) [23, 24] with 10-fold cross-validation. Baseline parameters were assigned penalty factors of 0 (to force inclusion), while other chromatin features were assigned penalty factors of 1. Features and coefficients were taken from the λ_se_. In addition to 3 baseline features, there were 38 features included in our platelet model and 78 features in our red cell model. Only the chromatin features and related coefficients were carried forward for model applications. For downstream genome-wide analyses, we scored all SNPs within NCBI dbSNP Build 147 based on coefficients and overlaps with model features.

### Model performance comparison

We used public databases to obtain SNP scores for alternative models (CADD v1.3 [29], GWAVA unmatched score [17], DeepSEA [28]; https://cadd.gs.washington.edu/download, http://www.sanger.ac.uk/resources/software/gwava, http://deepsea.princeton.edu). For each model, we identified scores for platelet trait GWAS SNPs and a random selection of ~ 100 control SNPs for each GWAS SNP. We then used ROCR [70] to compare model performance in discriminating GWAS SNPs from controls, and report the area under the receiver operating characteristic (AUC) for each model. An analogous pipeline was used to analyze the ability of each model to discriminate red cell trait-related GWAS SNPs from controls.

For sites in Table 1, including rs11071720, we obtained gChromVAR scores [1] (https://molpath.shinyapps.io/ShinyHeme/).

### Model evaluation

To assess biological specificity, we identified the top 1% highest-scoring SNPs from each model (platelet model, red cell model, GWAVA, CADD) after excluding all red cell or platelet trait-associated GWAS loci. We then used closestBed (https://bedtools.readthedocs.io/en/latest/content/tools/closest.html) to identify the nearest gene to each of these SNPs. Genes and position were defined by BioMart (http://www.biomart.org/). We then used the Gene Ontology resource (http://geneontology.org/) to analyze pathway enrichment. Input analysis settings were binomial tests and calculated FDR for GO Biological Process complete. Pathways identified with FDR < 5% are presented in Fig. 2d, Additional file 1: Table S3-S5, Additional file 2: Figure S16d, and Additional file 1: Table S8.

Enhancer regulatory regions were defined according to the FANTOM5 dataset [34]. Presented FANTOM5 data represent scores for all overlapping SNPs from dbSNP 147.

### Linkage disequilibrium structure assessment

The SNP Annotation and Proxy Search tool (https://archive.broadinstitute.org/mpg/snap/ldsearch.php), LDlink (https://analysistools.nci.nih.gov/LDlink), and 1000 Genomes Project (phase 3) data were used to measure linkage disequilibrium in the EUR population.

### Transcription factor binding site identification

To identify GATA sites, the genomic sequence context for SNPs of high interest were obtained using the UCSC Table Browser [71] and analyzed for matches by manual curation of canonical or near-canonical GATA binding motif in all orientations (AGATAA, TTATCA, AATAGA, TTATCT; GATAA, AATAG, CTATT, TTATC).

### Human iPSC generation

iPSC models were generated as described from peripheral blood mononuclear cells [72]. The “CHOP10” and “CHOP14” lines were used in this study. CRISPR/Cas9-mediated genome editing was performed as described [73] per protocols from the CHOP Human Pluripotent Stem Cell Core Facility (https://ccmt.research.chop.edu/cores_hpsc.php) with the following guide sequences: 5′ (1) ATGACGAAAGGTACCACGTCAGG, 5′ (2) TGAGTACTGATGAAACTATCAGG, 3′ (1) CCCTTTTCTTGCTGCTGTGTTGG, and 3′ (2) GGAGAGTGATCAAGAAATGGAGG.

### Karyotype analysis

Chromosomal G-band analyses were performed by Cell Line Genetics (Madison, WI).

### Copy number variation analysis

Copy number variation (CNV) analysis was performed with the Children’s Hospital of Philadelphia Center for Applied Genomics. CNVs were called using PennCNV [74] based on an Illumina Infinium GSAMD-24v2-0 (hg19) microarray with 759,993 SNPs.

### iPSC hematopoietic differentiation and analysis

iPS cell cultures and primitive hematopoietic differentiations were performed as per published protocols [58, 75–77]. iPS cells were maintained on irradiated mouse embryonic feeder cells in human embryonic stem cell (ESC) medium (DMEM/F12 with 20% knockout serum, 100 μM non-essential amino acids, 0.075% sodium bicarbonate, 1 mM sodium pyruvate, 2 mM glutamine, 50 U/ml penicillin, 50 g/ml streptomycin (all from Invitrogen), 10–4 M β–mercaptoethanol (Sigma, St. Louis, MO), and 10 ng/ml human bFGF (Stemgent)). Medium was changed at least every 2 days, and colony clusters passaged weekly to new feeders ESC medium containing ROCK inhibitor (10 μM) using TrypLE (Invitrogen) and gentle scraping.

About 1 week prior to differentiation, iPSCs were transitioned to a “feeder-free” state by culturing on Matrigel-coated wells (BD Biosciences; 6-well tissue culture plate, Falcon 3046) in ESC medium under atmospheric O_2_ conditions.

Throughout hematopoietic differentiation, cells were maintained at 37 °C in 5% CO_2_, 5% O_2_, and 90% N_2_. All media were supplemented with 2 mM glutamine, 50 μg/ml ascorbic acid (Sigma, St. Louis, MO), 150 μg/ml transferrin (Roche Diagnostics), and 4 × 10^−4^ M monothioglycerol (Sigma). Media and cytokines were changed daily as follows [78]: days 0–1 RPMI (Invitrogen) with 5 ng/ml BMP4, 50 ng/ml VEGF, and 25 ng/ml Wnt3a; day 2 RPMI with 5 ng/ml BMP4, 50 ng/ml VEGF, and 20 ng/ml bFGF; day 3 SP34 (Invitrogen) with 5 ng/ml BMP4, 50 ng/ml VEGF, and 20 ng/ml bFGF; days 4–5 SP34 with 15 ng/ml VEGF and 5 ng/ml bFGF; day 6 serum-free differentiation medium (SFD) with 50 ng/ml VEGF, 100 ng/ml bFGF, 100 ng/ml SCF, and 25 ng/ml Flt3L; and days 7–9 SFD with 50 ng/ml VEGF, 100 ng/ml bFGF, 100 ng/ml SCF, 25 ng/ml Flt3L, 50 ng/ml TPO, 10 ng/ml IL-6, and 0.05–2 U EPO. In all differentiations, marked cell death occurred through day 2, after which time surviving cells formed an adherent monolayer. Analyses during differentiation therefore used 0.25% trypsin-EDTA (ThermoFisher Scientific; 1 ml/well, 5 min at room temperature) to dissociate monolayer cells.

By days 6–7, non-adherent floating hematopoietic progenitor cells (HPCs) appeared. HPCs were collected on days 7–9 and either frozen or used directly for further culture and/or analyses. HPCs cultured in 50 ng/ml thrombopoietin and 25 ng/ml SCF to generate megakaryocytes, 2 U erythropoietin and 25 ng/ml SCF to generate erythroid cells, or 200 ng/ml granulocyte/macrophage colony stimulating factor to generate myeloid cells.

Flow cytometry gating strategies for pluripotency (SSEA3^+^/SSEA4^+^), hemogenic endothelium (KDR^+^/CD31^+^), hematopoietic progenitors (CD43^+^ and CD41^+^/CD235^+^), and terminal lineages have been previously validated [58, 75–77].

### Flow cytometry

Flow cytometry analysis was performed on a Cytoflex LX, and FACS sorting was performed on a FACS Aria II (BD Biosciences). Flow cytometry data were analyzed using FlowJo 10 (Tree Star, Inc.). The following antibodies were used for flow cytometry: FITC-conjugated anti-CD41 (BioLegend), PE-conjugated anti-CD42b (BD Biosciences), APC-conjugated anti-CD235 (BD Biosciences), PB450-conjugated anti-CD45 (BioLegend), AF488-conjugated anti-SSEA3 (BioLegend), AF647-conjugated anti-SSEA4 (BioLegend), PE-conjugated anti-KDR (R&D Systems), PECy7-conjugated antiCD31 (BioLegend), PECy7-conjugated anti-CD34 (eBioscience), and FITC-conjugated anti-CD43 (BioLegend).

### Gene expression analysis by RT-semiquantitative PCR

Total RNA was prepared using PureLink RNA micro kits (Invitrogen) in which samples were treated with RNase-free DNase. The reverse transcription of RNA (100 ng–1 μg) into cDNA was performed using random hexamers with Superscript II Reverse Transcriptase (RT) (Life Technologies), according to the manufacturer’s instructions. Real-time quantitative polymerase chain reaction (PCR) was performed on QuantStudio 5 Real-Time PCR Instrument (Applied Biosystems). All experiments were done in triplicate with SYBR-GreenER pPCR SuperMix (Life Technologies), according to the manufacturer’s instructions. Primers (Additional file 1: Table S11) were prepared by Integrated DNA Technologies or Sigma Aldrich. Dilutions of human genomic DNA standards ranging from 100 ng/μl to 10 pg/μl were used to evaluate PCR efficiency of each gene relative to the housekeeping gene *TATA-Box Binding Protein* (*TBP*).

### Microarray analysis

For microarray analysis, 50,000 cells were FACS-sorted directly into TRIzol. RNA was extracted from using a miRNeasy Mini Protocol (Qiagen). Samples passing quality control were analyzed using the human Clariom D Assay (ThermoFisher Scientific) and analyzed using Transcriptome Analysis Console (ThermoFisher Scientific) Software and Gene Set Enrichment Analysis (http://software.broadinstitute.org/gsea/index.jsp) software.

### Cell analysis and imaging

For Cytospins, FACS-sorted MKs were spun onto a glass slide and stained with May-Grünwald and Giemsa. Images were obtained on an Olympus BX60 microscope with a × 40 objective. An Invitrogen EVOS microscope with a × 10 objective was used to image cells in culture.

### Western blots

Cell pellets were resuspended in Laemmli buffer, sonicated for 5 min, and boiled for 5 min at 95 °C. Lysates were centrifuged at 10,000 rpm for 5 min at room temperature, and supernatants were used for analysis. Lysate volumes were normalized to cell counts. Samples were run on 4–12% NuPAGE Bis-Tris gels (Invitrogen) and transferred onto nitrocellulose membranes (0.45um pore size, Invitrogen) at 350 mA for 90 min. Following blocking in 5% milk for 1 h, membranes were incubated with primary antibodies overnight at 4 °C. After washing thrice in TBST, membranes were incubated with secondary horseradish peroxidase-conjugate antibodies for 1 h at room temperature, washed in TBST thrice, and developed using ECL western blotting substrate (Pierce) and HyBlot CL autoradiography film (Denville Scientific). The following antibodies were used for western blotting: Rabbit anti-TPM1 (D12H4, #3910, Cell Signaling Technologies), Mouse anti-TPM1/TPM2 (15D12.2, MAB2254, Millipore Sigma), Mouse anti-TPM3 (3D5AH3AB4, ab113692, Abcam), Rabbit anti-TPM4 (AB5449, Millipore Sigma), and Mouse anti-β Actin (A1978, Sigma). Western blot band quantitation was performed using FIJI [79] (https://fiji.sc/).

### MK activation assay

MKs were pelleted and resuspended in Tyrode’s Salts (Sigma) with 0.1% bovine serum albumin (BSA) containing FITC-conjugated PAC-1 (BD Biosciences), PacBlue-conjugated CD42a (eBioscience), and APC-conjugated CD42b (eBioscience) at a concentration of roughly 100,000 cells per 50 μl. Following addition of Convulxin (Enzo Biochem) or Thrombin (Sigma), cells were incubated at room temperature in the dark for 10 min. Cells were then incubated on ice for 10 min. An additional 100 μl Tyrode’s Salts containing 0.1% BSA was added, and cells were immediately analyzed by flow cytometry.

### Data presentation

Genome-wide SNP scores were loaded as custom tracks into the UCSC Genome Browser [71]. Images depicting genomic loci were generated using this tool, as well as Gviz [80]. Other data were created and presented using R, Adobe Illustrator CS6, or GraphPad Prism 6.

### Statistics

Statistical analyses were conducted using R or GraphPad Prism 6.

### Data availability

All materials, data, code, and associated protocols will be promptly available to readers upon request.

## Supplementary information

**Additional file 1:Table S1.** The 860 chromatin feature tracks included in our LASSO analysis. These data were obtained from ENCODE [68], ChromHMM [81], and analyses of primary human MK cells [26]. **Table S2.** Chromatin features and coefficients comprising our penalized regression-based platelet scoring model. Coefficients for background parameters are included at the bottom of this list, but were not included in subsequent genome-wide SNP scoring. **Table S3.** Gene Ontology pathways that were significantly enriched in the top 1% of SNPs, as defined by platelet model scores. Presented pathways had false discovery rate (FDR) < 5%. **Table S4.** Gene Ontology pathways that were significantly enriched in the top 1% of SNPs, as defined by GWAVA scores. Presented pathways had false discovery rate (FDR) < 5%. **Table S5.** Gene Ontology pathways that were significantly enriched in the top 1% of SNPs, as defined by CADD scores. Presented pathways had false discovery rate (FDR) < 5%. **Table S6.** CRISPR/Cas9-edited *Tropomyosin 1* knockout (KO) iPSC lines did not incur any additional CNVs compared to the parent line. Analyses of wild type CHOP14 and CHOP10 ‘parent’ lines, and derivative *TPM1KO* ‘child’ lines, are shown. Karyotype and copy number variation (CNV) analyses for all child lines were consistent with parental iPSC lines. **Table S7.** Dysregulated molecular pathways in *TPM1KO* MKs. FACS-sorted MKs were analyzed by microarray, and gene set enrichment was performed. Upregulated Gene Ontology [30] pathways with FDR<25% are shown. There were no significantly downregulated pathways. GO, Gene Ontology. NES, nominal enrichment score. FDR, false discovery rate. **Table S8.** Chromatin features and coefficients comprising our penalized regression-based red cell scoring model. Coefficients for background parameters are included at the bottom of this list, but were not included in subsequent genome-wide SNP scoring. **Table S9.** Gene Ontology pathways that were significantly enriched in the top 1% of SNPs, as defined by red cell model scores. Presented pathways had false discovery rate (FDR) < 5%. **Table S10.** Penalized regression-based fine-mapping identifies eQTLs in established platelet and/or red cell trait GWAS loci that overlie GATA binding sites. Listed SNPs are within platelet or red cell trait GWAS LD blocks (EUR r^2^>0.7), scored in the top 5% by *both* our platelet trait and red cell models, overlap canonical or near-canonical GATA binding sites, and are eQTLs for at least 1 gene [41] (GTEx V7). Associated GWAVA [17] scores are present, if available. SNP rsIDs and locations refer to hg19 genome. **Table S11.** Semi-quantitative RT-PCR primers used in this study.

**Additional file 2: Figure S1.** Penalized regression identifies epigenetic features that discriminate platelet trait GWAS SNPs from matched controls. Area under the receiver operator curve (AUC) for platelet trait model. Penalized regression results depicting the regularization parameter (λ) vs. AUC. Top axis shows how many features were identified at each level of λ. Variation in AUC at each λ reflects 10-fold cross-validation. The λ_min_ (model with maximal AUC) and λ_se_ (minimal feature inclusion with AUC within 1 standard error of λ_min_) are shown, with λ_se_ model incorporating the indicated number of features. The final model, with 41 total features, included 38 chromatin features and 3 background characteristics (Distance to Nearest Gene, Minor Allele Frequency, and Number of SNPs in linkage disequilibrium). The AUC at λ_se_ was 0.726. Note that this AUC includes background characteristics, which were not used in subsequent genome-wide SNP score applications. **Figure S2.** High SNP scores for platelet trait model capture information from sub-genome-wide significant loci. **a,b** Higher SNP scores correlate with lower GWAS *p*-values for variation in **a** mean platelet volume (MPV) or **b** platelet count (PLT). SNPs were scored genome-wide and plotted against arbitrarily binned –log_10_(p-value) GWAS MPV or PLT variation values. A value of 7.3 for –log_10_(p-value) correlates with a *p*-value of 5x10^-8^. Box-and-whisker plots show 25th-to-75th percent interval (box) and standard deviation (whiskers). ****p < 0.0001 vs Column 1 (ANOVA, Dunnett’s multiple comparison test). Significant linear correlations existed between higher values of –log_10_(p-value) and SNP scores (Pr(>|t|)<2e-16 by linear regression significance test). **c,d** SNPs that nearly missed genome-wide significance for **c** MPV or **d** PLT were enriched for high SNP scores. SNPs that did not meet genome-wide significance were stratified into non-significant (*p*-value >0.05) and marginally significant (*p*-value between 5x10^-8^ and 0.05). Bars represent mean±SEM. ****p < 0.0001 by Wilcoxon Rank Sum test. **Figure S3.** Platelet trait model gives high scores to SNPs marking hematopoietic enhancer regions. Hematopoietic enhancer regions are enriched for high SNP scores based on our platelet trait model. FANTOM5-defined enhancer regions for adult bone marrow (BM) CD34+ (CNhs12553), K562 (human erythroleukemia, CNhs12458), and CMK (human megakaryoblastic leukemia, CNhs11859) hematopoietic cells were compared with enhancer regions from random non-relevant cell types (CNhs11756 from adult pancreas, CNhs14245 from a papillary cell lung adenocarcinoma cell line and CNhs12849 from adult parotid gland). Bars represent mean±SEM. ****p<0.0001 by 1-way ANOVA vs Controls. **Figure S4.** Additional putatively active eQTLs implicated through fine-mapping with LASSO-based SNP scores and by direct overlap with GATA binding sites. In each panel, the top portion shows GWAS SNP in black and linked SNPs (EUR *r*^2^>0.7) in cyan. Bar heights depict SNP scores. Gene exons are shown in yellow. Accessible chromatin regions (ATAC-Seq) are shown for hematopoietic stem cells (HSC), CD34+ hematopoietic progenitor cells, megakaryocyte-erythroid progenitors (MEP), and erythroblasts (Ery) [53]. Implicated SNP(s) in each region are outlined in the gray box, and interesting gene(s) in each region are indicated. Note that some SNPs regulate multiple genes, but only nearby regulated genes are boxed and labeled here. **a** rs11240368 is an eQTL for *CNTN2* and *TMEM81*. **b** rs3771535 is an eQTL for *GMCL1* and *SNRNP27*. **c** rs10180681 and rs10180682 are eQTLs for *EPB41L5, PTPN4,* and *RALB*. **d** rs9646785 is an eQTL for *GAD1* and *GORASP2*. **e** rs6771578 is an eQTL for *PDCD10, SERPINI1,* and *WDR49*. **f** rs12652692 is an eQTL for *LHFPL2* and *SCAMP1*. **g** rs72793280 is an eQTL for *ACSL6, P4HA2, PDLIM4, SLC22A4,* and *SLC22A5*. **h** rs1741820 is an eQTL for *HSF2* and *PKIB*. **i** rs13265995 is an eQTL for *LYN, TGS,* and *TMEM68.***j** rs9704108 is an eQTL for *IFITM2.***k** rs2316513 is an eQTL for *DPH1, SMG6,* and *SRR.***l** rs1654439 is an eQTL for *GP6, NLRP2,* and *RDH13.* Scale bars, 50 kb. **Figure S5.** The SNP rs11071720 is an expression quantitative trait locus (eQTL) for *TPM1*. Individuals with the rs11071720 minor ‘C’ allele have decreased *Tropomyosin 1* expression in tibial artery tissue (*p*= 0.000056, Normalized Enrichment Score= -0.082). Data obtained from GTEx V7 [41]. **Figure S6.** DNA sequencing and western blot confirmation of *TPM1* deletion. **a** Shown are *TPM1* exons (numbered light blue boxes) in and around the proposed deletion site. 5’ and 3’ guide RNA sites are marked. Deleted areas in each clone are indicated as ‘empty’ bars, with flanking present DNA in dark red. **b** Western blot of CHOP14 or CHOP10 iPSC lysates showing no TPM1 protein in KO clones. Middle lane in CHOP10 blot depicts a suspected heterozygous clone. **Figure S7.** Karyotype analyses of iPSC clones were normal. **a,b,c** Analyses of **a** wild type CHOP14 performed at the time of genome editing, **b** CHOP14-derived *TPM1* knockout clone 1 (KO1), and **c** CHOP14-derived *TPM1* knockout clone 2 (KO2) show normal human female karyotypes. **d,e** Analyses of **d** wild type CHOP10 karyotype analysis performed at the time of genome editing and **e** CHOP10-derived *TPM1* knockout clone (KO3) show normal human male karyotypes. These results reflect analyses and interpretations from Cell Line Genetics (Madison, WI). **Figure S8.** KO cells show normal kinetics of pluripotency marker loss in early differentiation. **a** Representative gating strategy for flow cytometry analysis. Singlet cells were analyzed directly for all presented studies. **b** On days 0-4, TPM1 KO iPSCs show normal loss of pluripotency markers SSEA3 and SSEA4, with kinetics identical to WT. **Figure S9.** CHOP10-derived *TPM1* KO iPSCs yield more single cells after differentiation. There were more hematopoietic progenitor cells (HPCs, non-adherent single cells) in CHOP10-derived *TPM1* KO clone 3 following 7-8 hematopoietic differentiation. **p<0.01. **Figure S10.** Non-adherent cells (HPCs) from *TPM1* KO cultures show normal cell surface markers. WT and *TPM1* KO iPSC clones 1-3 all display relatively normal cell surface marker patterns after 9 d differentiation. Multiple experiments show no consistent lineage preference across all clones. **Figure S11.***TPM1* KO MKs have normal morphology. Following 8 d differentiation and 5 d MK expansion culture, wild type (WT) and *TPM1* KO CD41^+^/CD42b^+^ primitive MKs were FACS-sorted and analyzed by Cytospin. Scale bar represents 20 μm. **Figure S12.** Microarray analysis shows no significant differences in MK genes. **a** Volcano plot showing gene expression changes in WT and KO MK microarray analysis. *TPM1* is circled. **b** Hierarchical clustering for microarray gene analysis data of FACS-sorted WT and KO MKs. Red, high expression. Blue, low expression. **c** Heat map shows the most highly upregulated (top) and downregulated (bottom) genes in KO MKs. **d** Expression of representative MK genes are not significantly (ns) changed in WT vs KO MKs. PF4, *Platelet factor 4*. PPBP, *Pro-platelet basic protein*. SELP, *P-selectin*. NFE2, *Nuclear factor erythroid 2*. **e** Gene set enrichment analysis (GSEA) for MK pathways were not significantly changed. Shown are GO pathways for MK differentiation (FDR q-value 0.314) and Regulation of MK differentiation (FDR q-value 0.64). **f** GSEA plots for select significantly upregulated pathways in KO MKs. **Figure S13.***TPM1KO* HPCs retain normal myeloid lineage expansion potential. HPCs obtained after 8d differentiation were put into lineage expansion media and cultures were analyzed by manual cell counting and flow cytometry over 3-5 d. Mature myeloid cells were CD45^+^. Points represent lineage-specific cell percentage multiplied by total cell count, normalized to cell count on day 0. *p<0.05 by ANOVA vs WT. **Figure S14.** Hematopoietic trait associations of SNPs near and within the *TPM1* gene locus. Aggregated GWAS platelet, red cell, or white cell trait *p*-values for SNPs near and within the *TPM1* gene locus in LD with rs11071720. The p-values for these SNPs reach genome-wide significance for platelet traits (PLT#, MPV). **Figure S15.** Penalized regression identifies epigenetic features that discriminate red blood cell trait GWAS SNPs from matched controls. **a** Area under the receiver operator curve (AUC) for red cell trait model. Penalized regression results depicting the regularization parameter (λ) vs. AUC. Top axis shows how many features were identified at each level of λ. Variation in AUC at each λ reflects 10-fold cross-validation. The λ_min_ (model with maximal AUC) and λ_se_ (minimal feature inclusion with AUC within 1 standard error of λ_min_) are shown. The λ_se_ model incorporated 81 total features, including background characteristics (Distance to Nearest Gene, Minor Allele Frequency, and Number of SNPs in linkage disequilibrium). The AUC at λ_se_ was 0.732, though it is important to note that this included background characteristics (distance to nearest gene, number of SNPs in linkage disequilibrium, and minor allele frequency). **b** Penalized regression (LASSO) analysis identified 78 chromatin features from the indicated cell types that best discriminated red cell GWAS SNPs, after controlling for background characteristics. Bar heights are LASSO coefficients, indicating the relative importance of each feature. Subsequent application of this model was based only on these 78 chromatin features and associated coefficients. Ery, peripheral blood derived erythroblasts. MK, primary megakaryocytes. MK/Ery, K562 cells. Lymphoblast, GM12878 or GM12891. **Figure S16.** Penalized regression model identifies genes relevant to erythroid and hematopoietic biology. **a** SNP scores for red cell trait model training SNPs, or a set of validation red cell trait GWAS SNPs, were significantly higher than genome-wide SNP scores. Bars represent mean±SEM, ****p<0.0001 by ANOVA. **b** Performance comparison of our red cell trait model to DeepSEA [28], GWAVA [17], and CADD [29] for training red cell GWAS SNP identification. AUC values are shown in the legend. **c** Performance comparison of the indicated methods for validation red cell GWAS SNP identification. AUC values are shown in the legend corresponding to model accuracy in predicting validation SNPs (LASSO *n*=152, GWAVA *n*=29, CADD *n*=23, DeepSEA n=152) vs. ~15,000 random controls. **d** Erythroid and hematopoiesis pathways [30] identified by the highest-scoring (top 1%) SNPs genome-wide for the red cell model, excluding established red cell trait loci [6] (FDR, False Discovery Rate).** Figure S17.** High SNP scores for red cell trait model capture information from sub-genome-wide significant loci. **a,b** Higher SNP scores correlate with lower GWAS *p*-values for variation in **a** mean corpuscular volume (MCV) or **b** red blood cell count (RBC). SNPs were scored genome-wide and plotted against arbitrarily binned –log_10_(*p*-value) GWAS MCV or RBC variation values. A value of 7.3 for –log_10_(*p*-value) correlates with a *p*-value of 5x10^-8^. Box-and-whisker plots show 25th-to-75th percent interval (red box) and standard deviation (whiskers). ****p < 0.0001 vs Column 1 (ANOVA, Dunnett’s multiple comparison test). Significant linear correlations existed between higher values of –log_10_(p-value) and SNP scores (Pr(>|t|)<2e-16 by linear regression significance test). **c,d** SNPs missed genome-wide significance for **c** MCV or **d** RBC were enriched for high SNP scores. SNPs that did not meet genome-wide significance were stratified into non-significant (*p*-value > 0.05) and marginally significant (*p*-value between 5x10^-8^ and 0.05). Bars represent mean±SEM. ****p < 0.0001 by Wilcoxon Rank Sum test.

## Data Availability

Relevant datasets and coding scripts can be found on GitHub (https://github.com/thomchr/2019.PLT.TPM1.Paper). All materials, data, code, or associated protocols will also be promptly available to readers upon request.
