# Supplementary material for: Tropomyosin 1 genetically constrains in vitro hematopoiesis
Source: BMC Biol. 2020 May 14;18:52. doi: 10.1186/s12915-020-00783-7 (PMC7227211; doi:10.1186/s12915-020-00783-7)
Supplement: Supplementary file 2 — Additional file 2: Figure S1. Penalized regression identifies epigenetic features that discriminate platelet trait GWAS SNPs from matched controls. Area under the receiver operator curve (AUC) for platelet trait model. Penalized regression results depicting the regularization parameter (λ) vs. AUC. Top axis shows how many features were identified at each level of λ. Variation in AUC at each λ reflects 10-fold cross-validation. The λmin (model with maximal AUC) and λse (minimal feature inclusion with AUC within 1 standard error of λmin) are shown, with λse model incorporating the indicated number of features. The final model, with 41 total features, included 38 chromatin features and 3 background characteristics (Distance to Nearest Gene, Minor Allele Frequency, and Number of SNPs in linkage disequilibrium). The AUC at λse was 0.726. Note that this AUC includes background characteristics, which were not used in subsequent genome-wide SNP score applications. Figure S2. High SNP scores for platelet trait model capture information from sub-genome-wide significant loci. a,b Higher SNP scores correlate with lower GWAS p-values for variation in a mean platelet volume (MPV) or b platelet count (PLT). SNPs were scored genome-wide and plotted against arbitrarily binned –log10(p-value) GWAS MPV or PLT variation values. A value of 7.3 for –log10(p-value) correlates with a p-value of 5x10^-8^. Box-and-whisker plots show 25th-to-75th percent interval (box) and standard deviation (whiskers). ****p < 0.0001 vs Column 1 (ANOVA, Dunnett’s multiple comparison test). Significant linear correlations existed between higher values of –log10(p-value) and SNP scores (Pr(>|t|)<2e-16 by linear regression significance test). c,d SNPs that nearly missed genome-wide significance for c MPV or d PLT were enriched for high SNP scores. SNPs that did not meet genome-wide significance were stratified into non-significant (p-value >0.05) and marginally significant (p-value between 5x10^-8^ and 0.05). Bars [file 12915_2020_783_MOESM2_ESM.pdf]

Tropomyosin 1 genetically constrains *in vitro* hematopoiesis

Thom CS[1,2,3]*, Jobaliya CD[4,5], Lorenz K[2,3], Maguire JA[4,5], Gagne A[4,5], Gadue P[4,5], French DL[4,5], Voight BF[2,3,6]*

[1]Division of Neonatology, Children's Hospital of Philadelphia, Philadelphia, PA, USA
[2]Department of Systems Pharmacology and Translational Therapeutics, Perelman School of Medicine, University of Pennsylvania, Philadelphia, PA, USA
[3]Department of Genetics, Perelman School of Medicine, University of Pennsylvania, Philadelphia, PA, USA
[4]Center for Cellular and Molecular Therapeutics, The Children's Hospital of Philadelphia, PA, USA
[5]Department of Pathology and Laboratory Medicine, The Children's Hospital of Philadelphia, University of Pennsylvania, Philadelphia, PA, USA
[6]Institute of Translational Medicine and Therapeutics, University of Pennsylvania, PA, USA
*Corresponding authors: thomc@email.chop.edu, bvoight@pennmedicine.upenn.edu

Supplementary Information

Supplementary Figures and Figure Legends

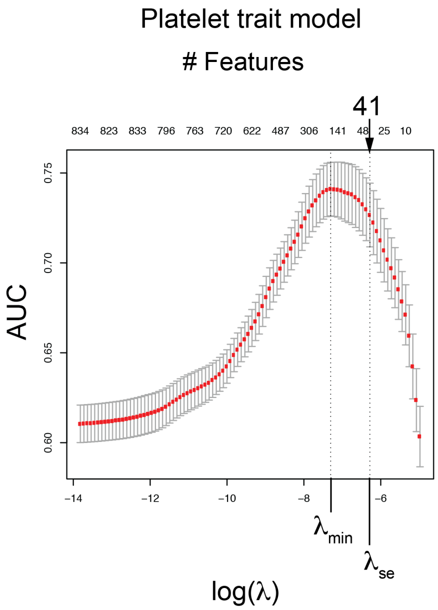

Figure S1. Penalized regression identifies epigenetic features that discriminate platelet trait GWAS SNPs from matched controls.

Area under the receiver operator curve (AUC) for platelet trait model. Penalized regression results depicting the regularization parameter (λ) vs. AUC. Top axis shows how many features were identified at each level of λ. Variation in AUC at each λ reflects 10-fold cross-validation. The λ_{min} (model with maximal AUC) and λ_{se} (minimal feature inclusion with AUC within 1 standard error of λ_{min}) are shown, with λ_{se} model incorporating the indicated number of features. The final model, with 41 total features, included 38 chromatin features and 3 background characteristics (Distance to Nearest Gene, Minor Allele Frequency, and Number of SNPs in linkage disequilibrium). The AUC at λ_{se} was 0.726. Note that this AUC includes background characteristics, which were not used in subsequent genome-wide SNP score applications.

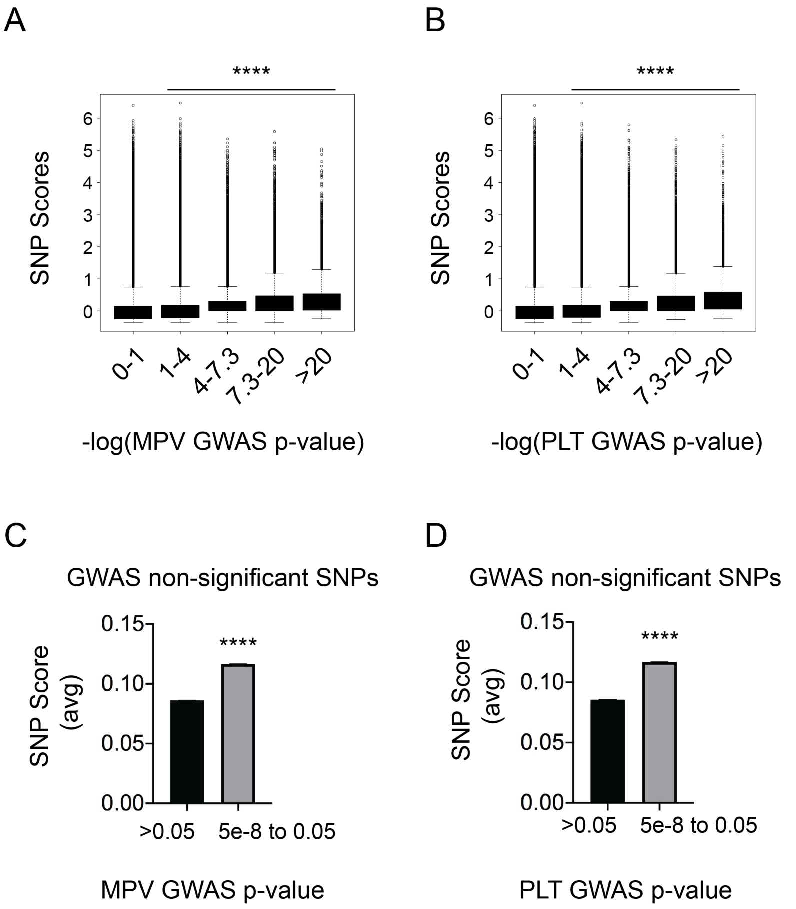

Figure S2. High SNP scores for platelet trait model capture information from sub-genome-wide significant loci.

a,b Higher SNP scores correlate with lower GWAS *p*-values for variation in **a** mean platelet volume (MPV) or **b** platelet count (PLT). SNPs were scored genome-wide and plotted against arbitrarily binned $-\log_{10}$(p-value) GWAS MPV or PLT variation values. A value of 7.3 for $-\log_{10}$(p-value) correlates with a *p*-value of 5×10^{-8}. Box-and-whisker plots show 25th-to-75th percent interval (box) and standard deviation (whiskers). ****p<0.0001 vs Column 1 (ANOVA, Dunnett's multiple comparison test). Significant linear correlations existed between higher values of $-\log_{10}$(p-value) and SNP scores (Pr(>|t|)<2e-16 by linear regression significance test).

c,d SNPs that nearly missed genome-wide significance for **c** MPV or **d** PLT were enriched for high SNP scores. SNPs that did not meet genome-wide significance were stratified into non-significant (*p*-value >0.05) and marginally significant (*p*-value between 5×10^{-8} and 0.05). Bars represent mean±SEM. ****p<0.0001 by Wilcoxon Rank Sum test.

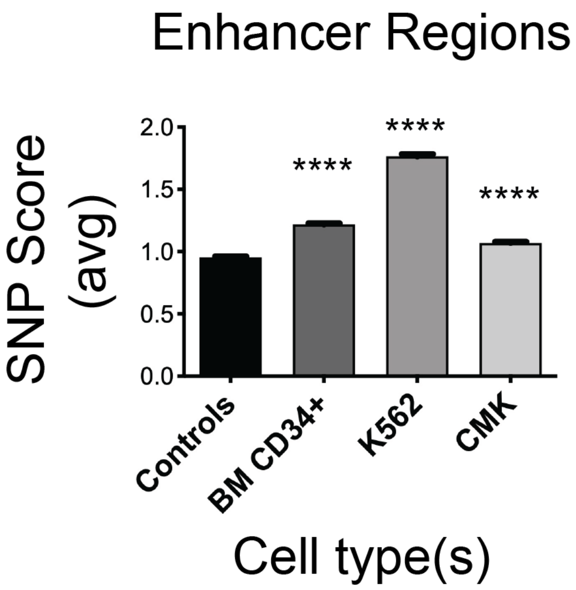

Figure S3. Platelet trait model gives high scores to SNPs marking hematopoietic enhancer regions.
Hematopoietic enhancer regions are enriched for high SNP scores based on our platelet trait model. FANTOM5-defined enhancer regions for adult bone marrow (BM) CD34+ (CNhs12553), K562 (human erythroleukemia, CNhs12458), and CMK (human megakaryoblastic leukemia, CNhs11859) hematopoietic cells were compared with enhancer regions from random non-relevant cell types (CNhs11756 from adult pancreas, CNhs14245 from a papillary cell lung adenocarcinoma cell line and CNhs12849 from adult parotid gland). Bars represent mean±SEM. *****p*<0.0001 by 1-way ANOVA vs Controls.

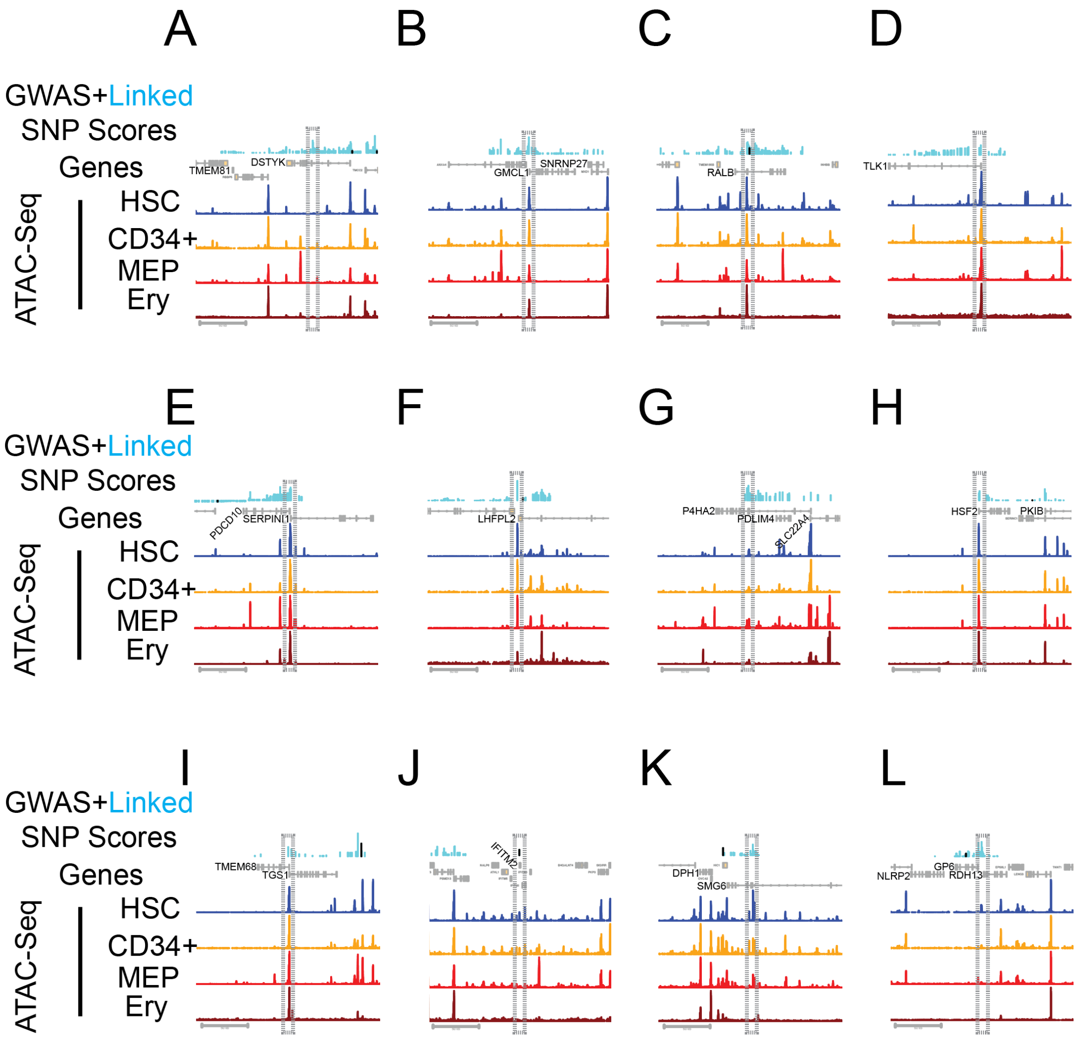

Figure S4. Additional putatively active eQTLs implicated through fine-mapping with LASSO-based SNP scores and by direct overlap with GATA binding sites. In each panel, the top portion shows GWAS SNP in black and linked SNPs (EUR r^2>0.7) in cyan. Bar heights depict SNP scores. Gene exons are shown in yellow. Accessible chromatin regions (ATAC-Seq) are shown for hematopoietic stem cells (HSC), CD34+ hematopoietic progenitor cells, megakaryocyte-erythroid progenitors (MEP), and erythroblasts (Ery) [39]. Implicated SNP(s) in each region are outlined in the gray box, and interesting gene(s) in each region are indicated. Note that some SNPs regulate multiple genes, but only nearby regulated genes are boxed and labeled here. **a** rs11240368 is an eQTL for *CNTN2* and *TMEM81*. **b** rs3771535 is an eQTL for *GMCL1* and *SNRNP27*. **c** rs10180681 and rs10180682 are eQTLs for *EPB41L5, PTPN4,* and *RALB*. **d** rs9646785 is an eQTL for *GAD1* and *GORASP2*. **e** rs6771578 is an eQTL for *PDCD10, SERPINI1,* and *WDR49*. **f** rs12652692 is an eQTL for *LHFPL2* and *SCAMP1*. **g** rs72793280 is an eQTL for *ACSL6, P4HA2, PDLIM4, SLC22A4,* and *SLC22A5*. **h** rs1741820 is an eQTL for *HSF2* and *PKIB*. **i** rs13265995 is an eQTL for *LYN, TGS,* and *TMEM68.* **j** rs9704108 is an eQTL for *IFITM2.* **k** rs2316513 is an eQTL for *DPH1, SMG6,* and *SRR.* **l** rs1654439 is an eQTL for *GP6, NLRP2,* and *RDH13.* Scale bars, 50 kb.

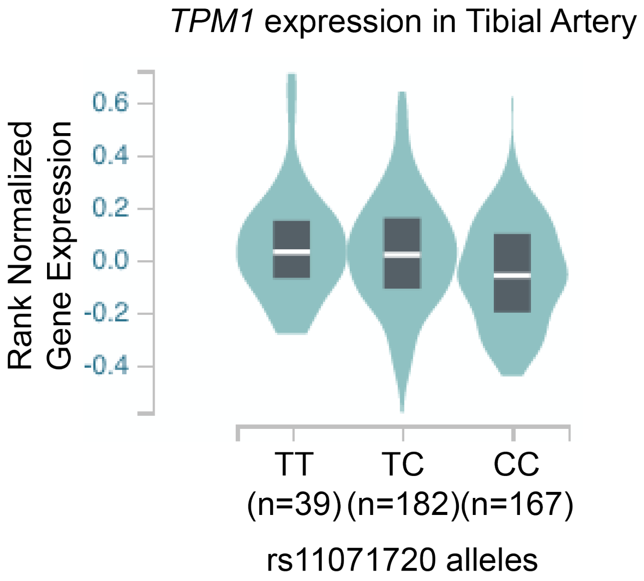

Figure S5. The SNP rs11071720 is an expression quantitative trait locus (eQTL) for *TPM1*.
Individuals with the rs11071720 minor ‘C’ allele have decreased *Tropomyosin 1* expression in tibial artery tissue (p= 0.000056, Normalized Enrichment Score= -0.082). Data obtained from GTEx V7 [43].

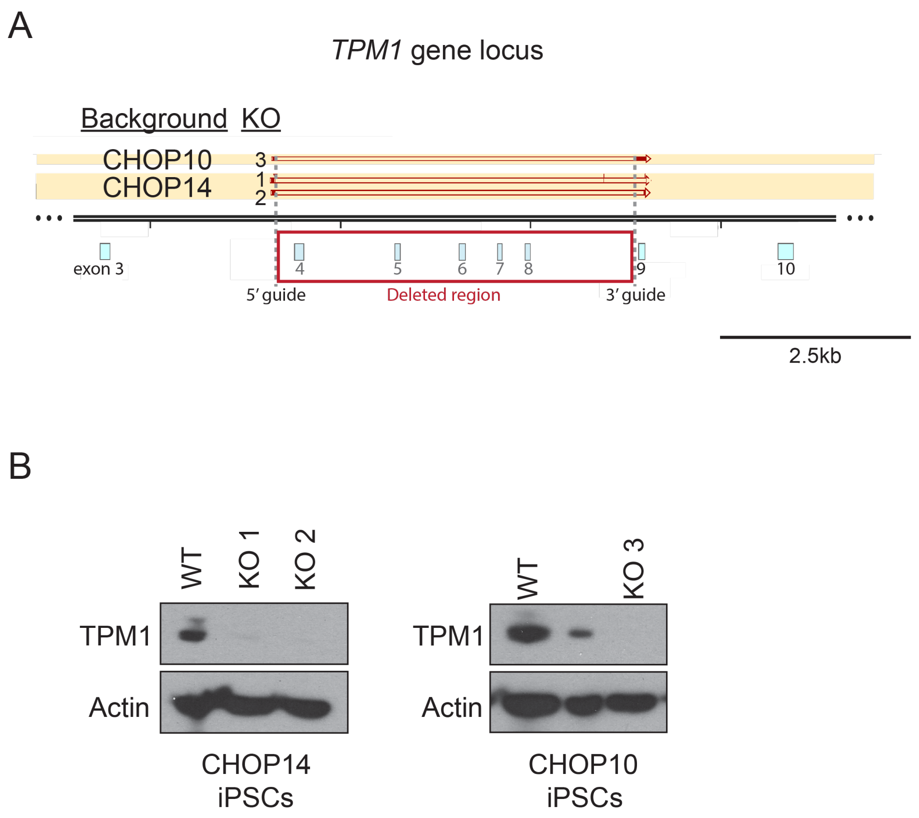

Figure S6. DNA sequencing and western blot confirmation of *TPM1* deletion.
a Shown are *TPM1* exons (numbered light blue boxes) in and around the proposed deletion site. 5' and 3' guide RNA sites are marked. Deleted areas in each clone are indicated as 'empty' bars, with flanking present DNA in dark red. **b** Western blot of CHOP14 or CHOP10 iPSC lysates showing no TPM1 protein in KO clones. Middle lane in CHOP10 blot depicts a suspected heterozygous clone.

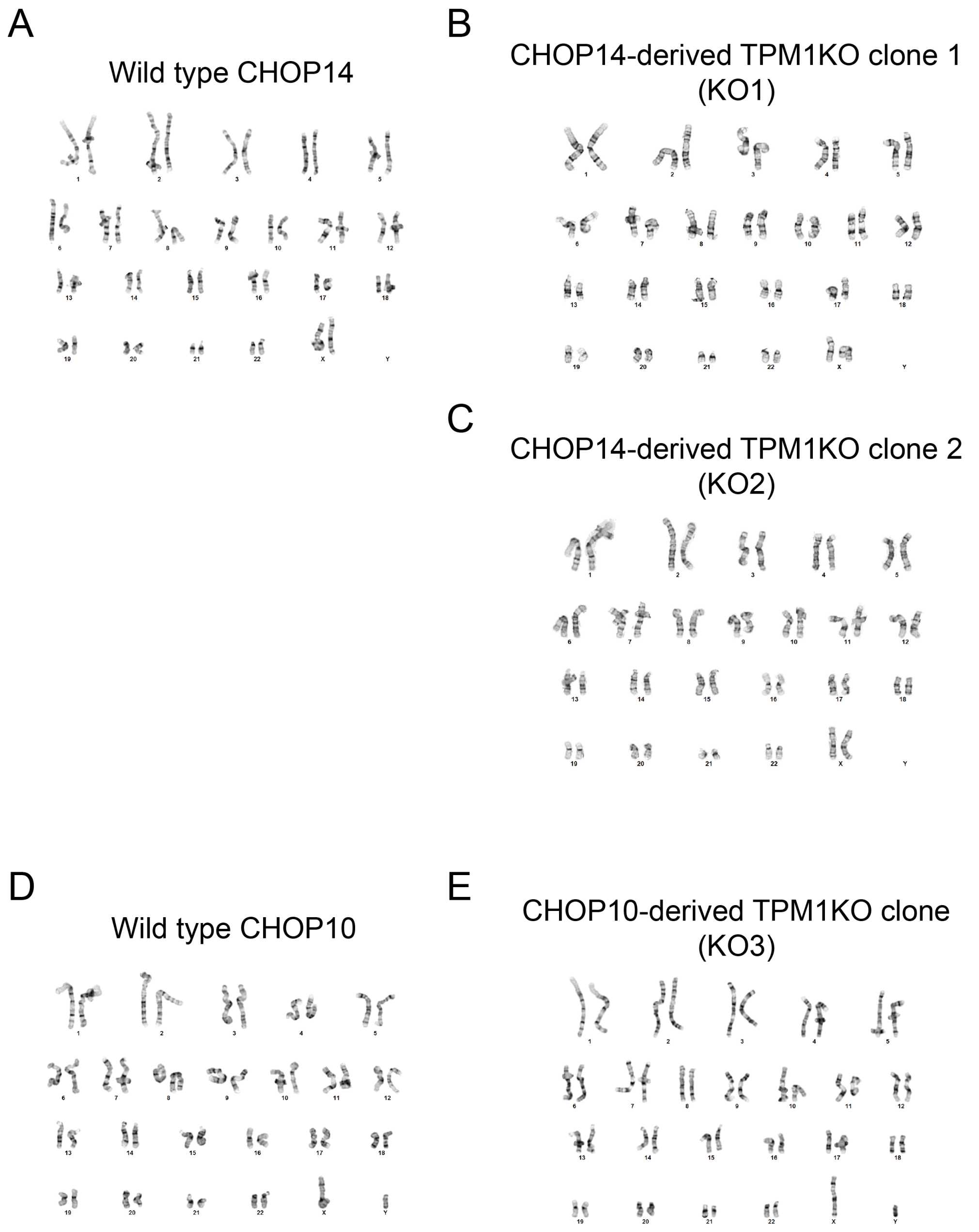

Figure S7. Karyotype analyses of iPSC clones were normal.
a,b,c Analyses of **a** wild type CHOP14 performed at the time of genome editing, **b** CHOP14-derived *TPM1* knockout clone 1 (KO1), and **c** CHOP14-derived *TPM1* knockout clone 2 (KO2) show normal human female karyotypes. **d,e** Analyses of **d** wild type CHOP10 karyotype analysis performed at the time of genome editing and **e** CHOP10-derived *TPM1* knockout clone (KO3) show normal human male karyotypes. These results reflect analyses and interpretations from Cell Line Genetics (Madison, WI).

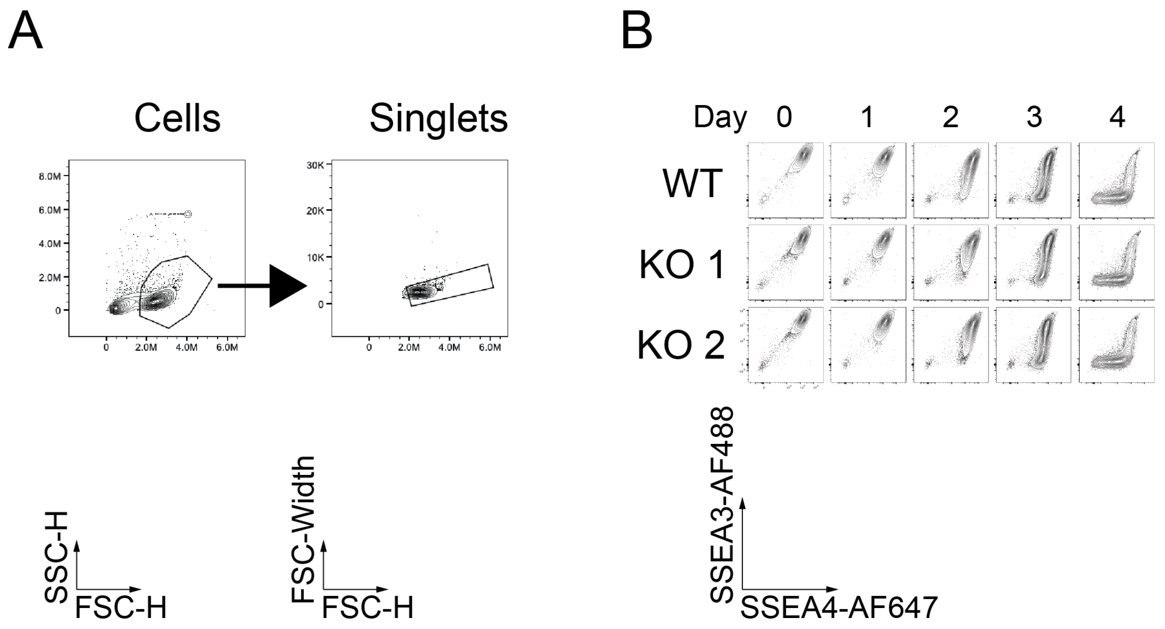

Figure S8. KO cells show normal kinetics of pluripotency marker loss in early differentiation.

a Representative gating strategy for flow cytometry analysis. Singlet cells were analyzed directly for all presented studies. **b** On days 0-4, TPM1 KO iPSCs show normal loss of pluripotency markers SSEA3 and SSEA4, with kinetics identical to WT.

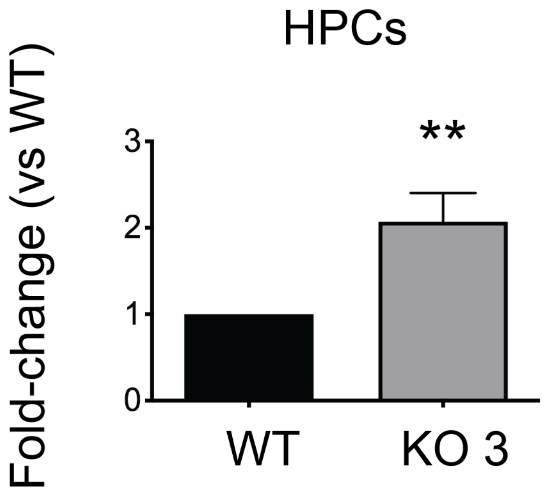

Figure S9. CHOP10-derived *TPM1* KO iPSCs yield more single cells after differentiation.
There were more hematopoietic progenitor cells (HPCs, non-adherent single cells) in CHOP10-derived *TPM1* KO clone 3 following 7-8 hematopoietic differentiation. ***p*<0.01.

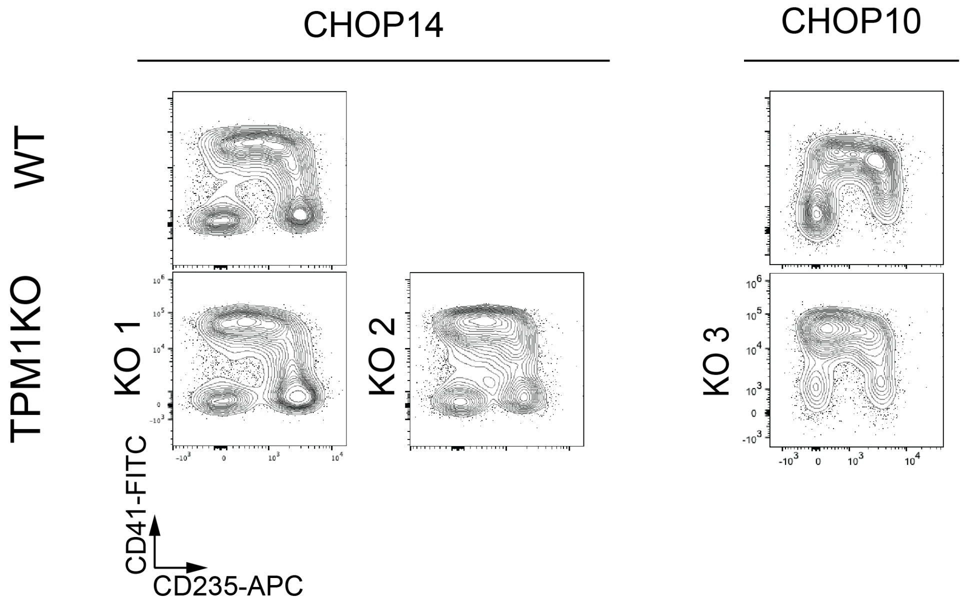

Figure S10. Non-adherent cells (HPCs) from *TPM1* KO cultures show normal cell surface markers.
WT and *TPM1* KO iPSC clones 1-3 all display relatively normal cell surface marker patterns after 9 d differentiation. Multiple experiments show no consistent lineage preference across all clones.

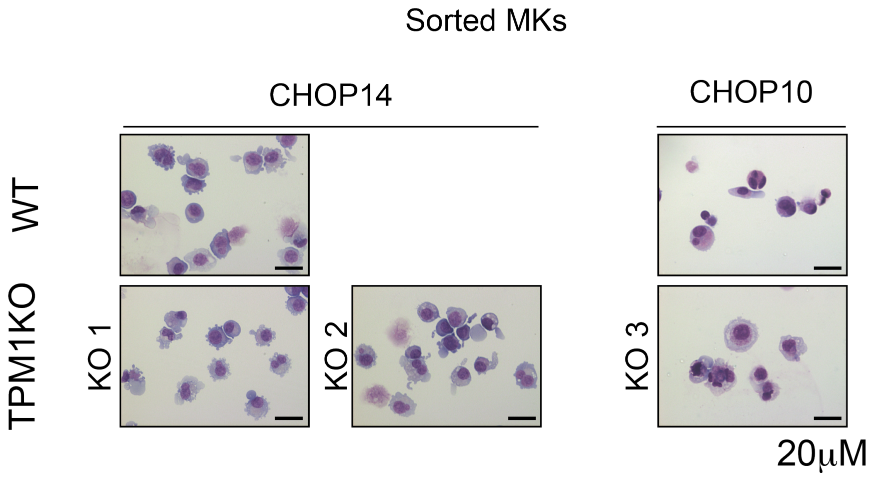

Figure S11. *TPM1* KO megakaryocytes (MKs) have normal morphology.
Following 8 d differentiation and 5 d MK expansion culture, wild type (WT) and *TPM1* KO CD41^{+}/CD42b^{+} primitive MKs were FACS-sorted and analyzed by Cytospin. Scale bar represents 20μm.

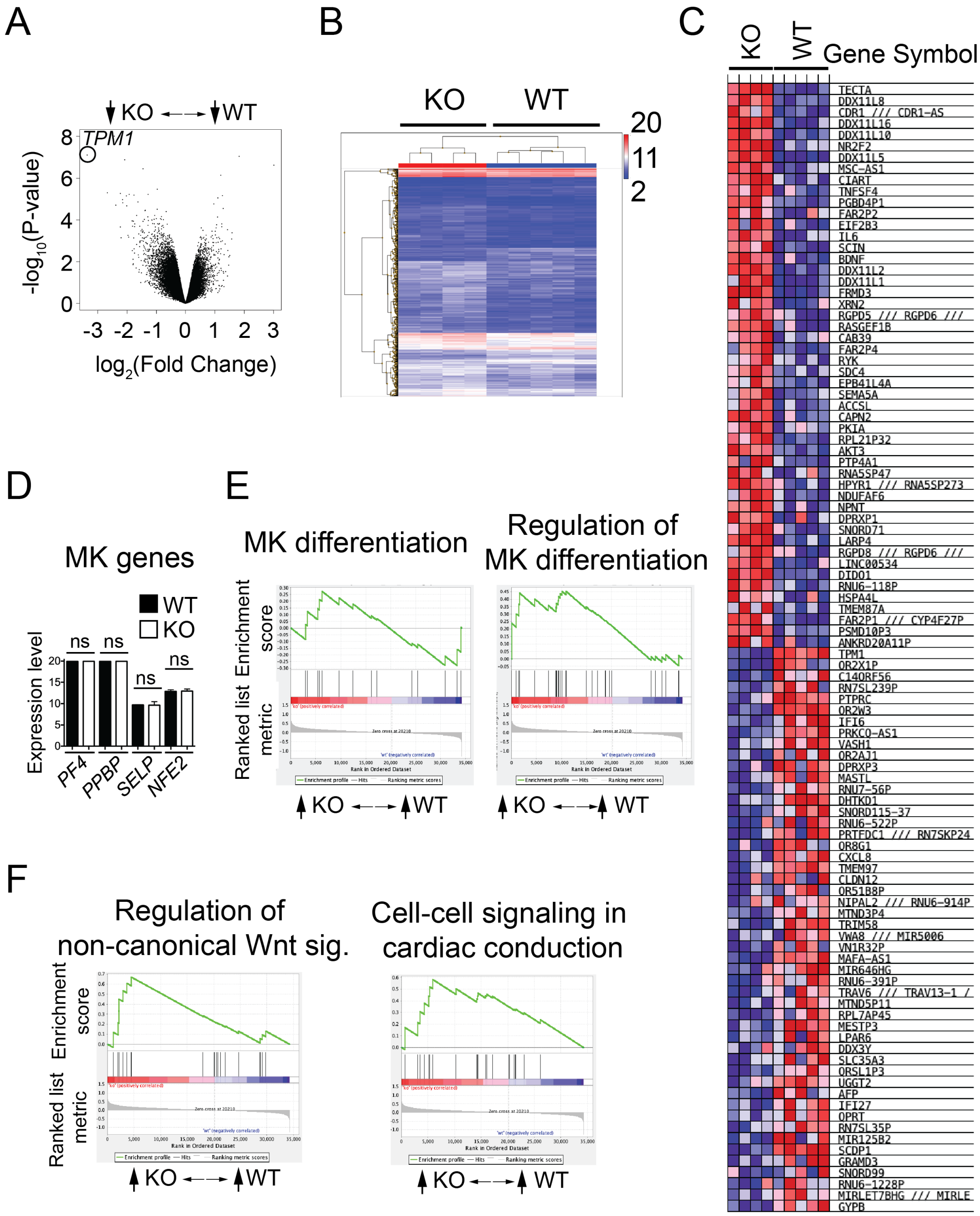

Figure S12. Microarray analysis shows no significant differences in MK genes. **a** Volcano plot showing gene expression changes in WT and KO MK microarray analysis. *TPM1* is circled. **b** Hierarchical clustering for microarray gene analysis data of FACS-sorted WT and KO MKs. Red, high expression. Blue, low expression. **c** Heat map shows the most highly upregulated (top) and downregulated (bottom) genes in KO MKs. **d** Expression of representative MK genes are not significantly (ns) changed in WT vs KO MKs. PF4, *Platelet factor 4*. PPBP, *Pro-platelet basic protein*. SELP, *P-selectin*. NFE2, *Nuclear factor erythroid 2*. **e** Gene set enrichment analysis (GSEA) for MK pathways were not significantly changed. Shown are GO pathways for MK differentiation (FDR q-value 0.314) and Regulation of MK differentiation (FDR q-value 0.64). **f** GSEA plots for select significantly upregulated pathways in KO MKs.

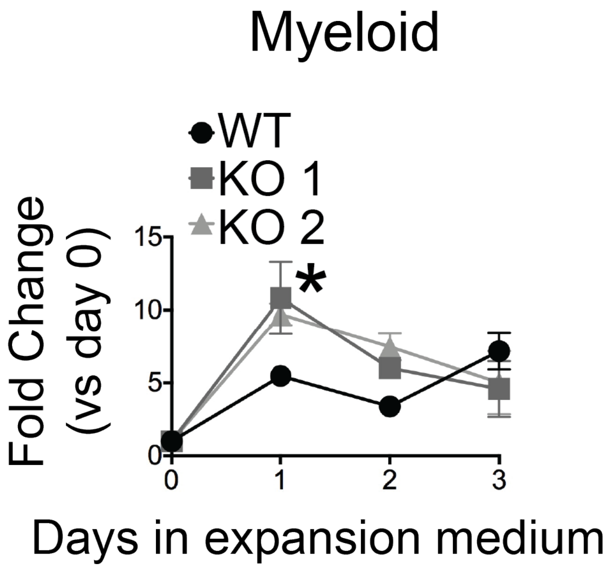

Figure S13. ***TPM1KO*** **HPCs retain normal myeloid lineage expansion potential.** HPCs obtained after 8 d differentiation were put into lineage expansion media and cultures were analyzed by manual cell counting and flow cytometry over 3-5 d. Mature myeloid cells were $CD45^+$. Points represent lineage-specific cell percentage multiplied by total cell count, normalized to cell count on day 0. **p*<0.05 by ANOVA vs WT.

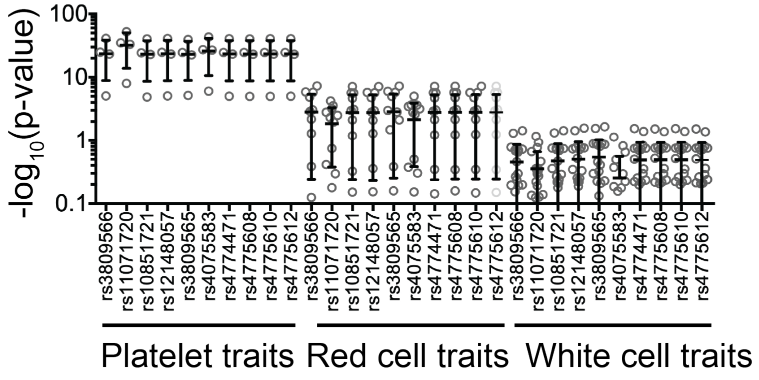

**Platelet traits**	**Red cell traits**	**White cell traits**
MPV	HCT	Baso
PCT	HGB	Baso_neut
PDW	HLR	Baso_p_gran
PLT#	HLR_p	Baso_p
	IRF	Eo
	MCHC	Eo_p
	MCH	Eo_baso
	MCV	Eo_p_gran
	RBC	Gran
	RDW	Gran_p_myeloid_wbc
	RET	Lymph
	RET_p	Lymph_p
		Mono
		Mono_p
		Myeloid_wbc
		Neut
		Neut_p
		Neut_eo
		Neut_p_gran
		WBC

Figure S14. Hematopoietic trait associations of SNPs near and within the *TPM1* gene locus.
Aggregated GWAS platelet, red cell, or white cell trait p-values for SNPs near and within the *TPM1* gene locus in LD with rs11071720. The *p*-values for these SNPs reach genome-wide significance for platelet traits (PLT#, MPV).

A

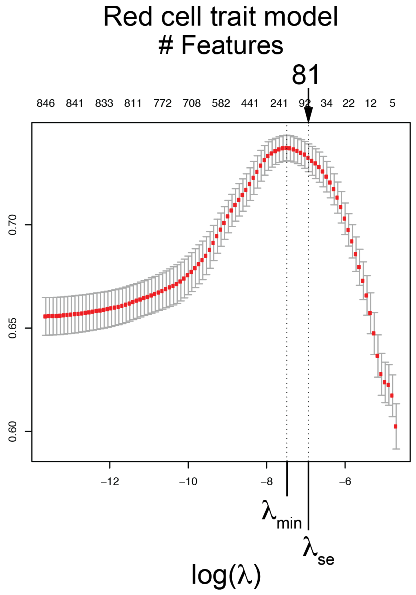

B

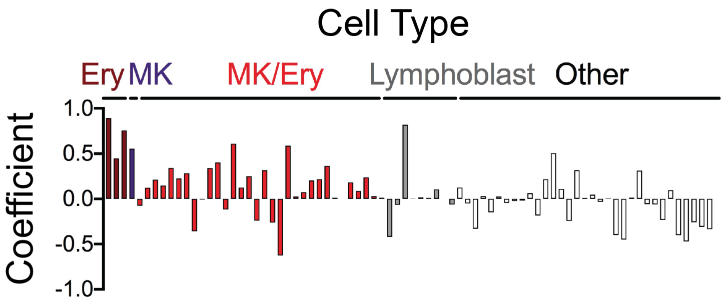

Figure S15. Penalized regression identifies epigenetic features that discriminate red blood cell trait GWAS SNPs from matched controls.

a Area under the receiver operator curve (AUC) for red cell trait model. Penalized regression results depicting the regularization parameter (λ) vs. AUC. Top axis shows how many features were identified at each level of λ. Variation in AUC at each λ reflects 10-fold cross-validation. The λ_{min} (model with maximal AUC) and λ_{se} (minimal feature inclusion with AUC within 1 standard error of λ_{min}) are shown. The λ_{se} model incorporated 81 total features, including background characteristics (Distance to Nearest Gene, Minor Allele Frequency, and Number of SNPs in linkage disequilibrium). The AUC at λ_{se} was 0.732, though it is important to note that this included background characteristics (distance to nearest gene, number of SNPs in linkage disequilibrium, and minor allele frequency). **b** Penalized regression (LASSO) analysis identified 78 chromatin features from the indicated cell types that best discriminated red cell GWAS SNPs, after controlling for background characteristics. Bar heights are LASSO coefficients, indicating the relative importance of each feature. Subsequent application of this model was based only on these 78 chromatin features and associated coefficients. Ery, peripheral blood derived erythroblasts. MK, primary megakaryocytes. MK/Ery, K562 cells. Lymphoblast, GM12878 or GM12891.

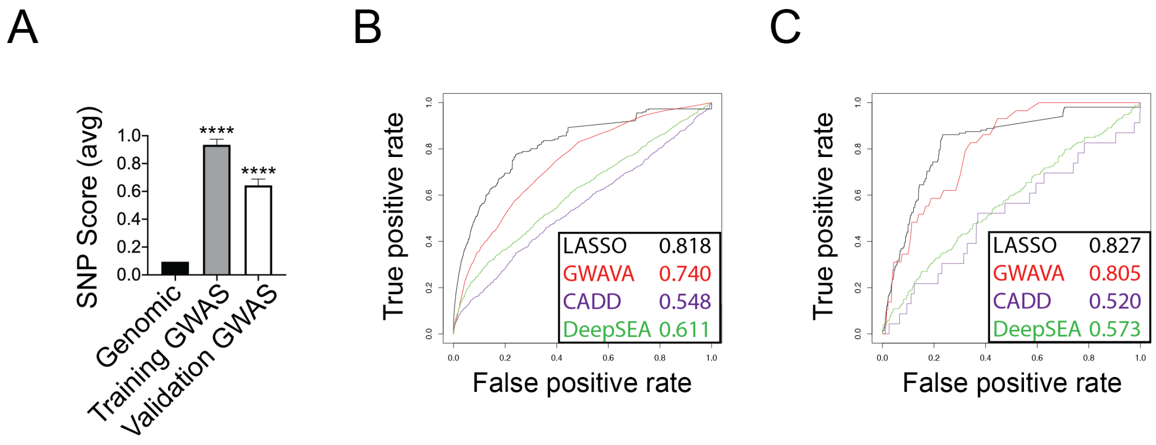

D

GO Biological Process	Fold Enrichment	FDR
**LASSO**		
erythrocyte differentiation (GO:0030218)	1.57	6.17E-03
erythrocyte homeostasis (GO:0034101)	1.56	4.04E-03
blood coagulation (GO:0007596)	1.27	1.16E-02
hemostasis (GO:0007599)	1.27	1.15E-02
coagulation (GO:0050817)	1.26	1.51E-02
regulation of hematopoietic progenitor cell differentiation (GO:1901532)	1.52	1.20E-02
regulation of hematopoietic stem cell differentiation (GO:1902036)	1.5	3.11E-02
regulation of hemopoiesis (GO:1903706)	1.29	1.83E-04
hemopoiesis (GO:0030097)	1.29	2.84E-05
hem. or lymph. organ dev (GO:0048534)	1.28	2.17E-05

Figure S16. Penalized regression model identifies genes relevant to erythroid and hematopoietic biology.
a SNP scores for red cell trait model training SNPs, or a set of validation red cell trait GWAS SNPs, were significantly higher than genome-wide SNP scores. Bars represent mean±SEM, *****p*<0.0001 by ANOVA. **b** Performance comparison of our red cell trait model to DeepSEA [28], GWAVA [17], and CADD [27] for training red cell GWAS SNP identification. AUC values are shown in the legend. **c** Performance comparison of the indicated methods for validation red cell GWAS SNP identification. AUC values are shown in the legend corresponding to model accuracy in predicting validation SNPs (LASSO n=152, GWAVA n=29, CADD n=23, DeepSEA n=152) vs. ~15,000 random controls. **d** Erythroid and hematopoiesis pathways [69] identified by the highest-scoring (top 1%) SNPs genome-wide for the red cell model, excluding established red cell trait loci [6] (FDR, False Discovery Rate).

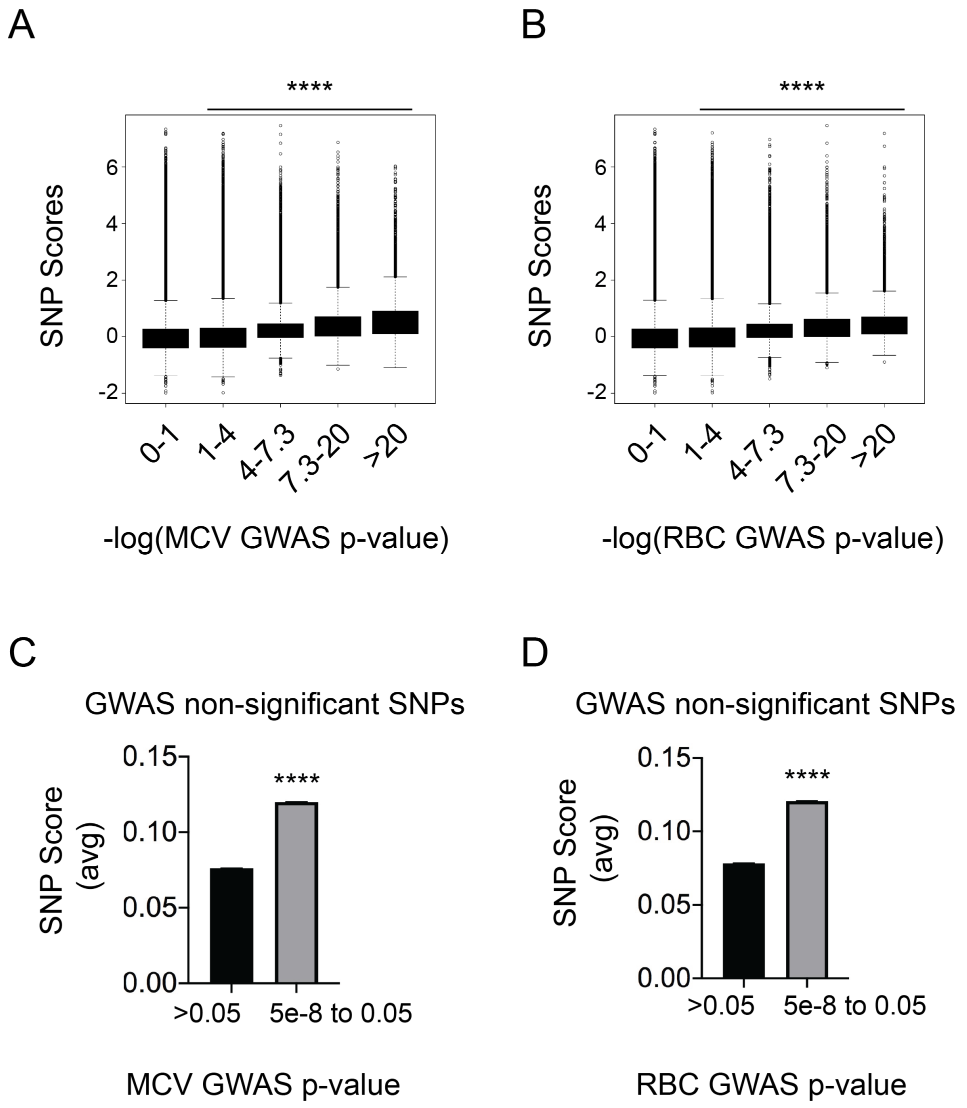

Figure S17. High SNP scores for red cell trait model capture information from sub-genome-wide significant loci.

a,b Higher SNP scores correlate with lower GWAS *p*-values for variation in **a** mean corpuscular volume (MCV) or **b** red blood cell count (RBC). SNPs were scored genome-wide and plotted against arbitrarily binned $-\log_{10}$(p-value) GWAS MCV or RBC variation values. A value of 7.3 for $-\log_{10}$(p-value) correlates with a *p*-value of 5×10^{-8}. Box-and-whisker plots show 25th-to-75th percent interval (red box) and standard deviation (whiskers). ****$p<0.0001$ vs Column 1 (ANOVA, Dunnett's multiple comparison test). Significant linear correlations existed between higher values of $-\log_{10}$(p-value) and SNP scores (Pr(>|t|)<2e-16 by linear regression significance test).

c,d SNPs missed genome-wide significance for **c** MCV or **d** RBC were enriched for high SNP scores. SNPs that did not meet genome-wide significance were stratified into non-significant (*p*-value >0.05) and marginally significant (*p*-value between 5×10^{-8} and 0.05). Bars represent mean±SEM. ****$p<0.0001$ by Wilcoxon Rank Sum test.
